# The Relative and Combined Effects of Noise Exposure and Aging on Auditory Peripheral Neural Deafferentation: A Narrative Review

**DOI:** 10.3389/fnagi.2022.877588

**Published:** 2022-06-23

**Authors:** Adnan M. Shehabi, Garreth Prendergast, Christopher J. Plack

**Affiliations:** ^1^Manchester Centre for Audiology and Deafness, University of Manchester, Manchester, United Kingdom; ^2^Department of Audiology and Speech Therapy, Birzeit University, Birzeit, Palestine; ^3^Department of Psychology, Lancaster University, Lancaster, United Kingdom

**Keywords:** cochlear synaptopathy (CS), noise exposure, age-related hearing loss (ARHL), auditory brainstem response (ABR), summating potential to action potential ratio (SP:AP), envelope-following response (EFR), middle ear muscle reflex (MEMR), speech-perception-in-noise (SPiN)

## Abstract

Animal studies have shown that noise exposure and aging cause a reduction in the number of synapses between low and medium spontaneous rate auditory nerve fibers and inner hair cells before outer hair cell deterioration. This noise-induced and age-related cochlear synaptopathy (CS) is hypothesized to compromise speech recognition at moderate-to-high suprathreshold levels in humans. This paper evaluates the evidence on the relative and combined effects of noise exposure and aging on CS, in both animals and humans, using histopathological and proxy measures. In animal studies, noise exposure seems to result in a higher proportion of CS (up to 70% synapse loss) compared to aging (up to 48% synapse loss). Following noise exposure, older animals, depending on their species, seem to either exhibit significant or little further synapse loss compared to their younger counterparts. In humans, temporal bone studies suggest a possible age- and noise-related auditory nerve fiber loss. Based on the animal data obtained from different species, we predict that noise exposure may accelerate age-related CS to at least some extent in humans. In animals, noise-induced and age-related CS in separation have been consistently associated with a decreased amplitude of wave 1 of the auditory brainstem response, reduced middle ear muscle reflex strength, and degraded temporal processing as demonstrated by lower amplitudes of the envelope following response. In humans, the individual effects of noise exposure and aging do not seem to translate clearly into deficits in electrophysiological, middle ear muscle reflex, and behavioral measures of CS. Moreover, the evidence on the combined effects of noise exposure and aging on peripheral neural deafferentation in humans using electrophysiological and behavioral measures is even more sparse and inconclusive. Further research is necessary to establish the individual and combined effects of CS in humans using temporal bone, objective, and behavioral measures.

## Introduction

Noise exposure during work and/or leisure activities is associated with a range of disorders including noise-induced hearing loss (NIHL), tinnitus, hyperacusis, temporary threshold shift, compromised sleep, increased stress, and hypertension (Concha-Barrientos et al., 2004; Nelson et al., 2005). The effect of aging on the human auditory system is often described as presbycusis or age-related hearing loss (ARHL); (Huang and Tang, 2010). In ARHL, peripheral and central auditory deterioration takes place which results in a wide variety of auditory symptoms including high-frequency sensorineural hearing loss, impaired sound localization, speech-perception-in-noise (SPiN) difficulties, poor central auditory processing, and impaired temporal processing (Mazelova et al., 2003; Gates and Mills, 2005; Jayakody et al., 2018). Although there is no agreement on a single etiology of ARHL, factors such as genetic predisposition, cumulative lifetime noise exposure, intake of ototoxic medications, and past auditory pathologies may be potential underlying causes (Gates and Mills, 2005; Dubno et al., 2013).

Excessive noise exposure and aging are both associated with major damage to cochlear outer hair cells (OHCs) and their stereocilia, with a lesser impact on inner hair cells (IHCs) (Wang et al., 2002; Gates and Mills, 2005; Popelar et al., 2006; Sergeyenko et al., 2013; Jayakody et al., 2018; Wu et al., 2021). This cochlear hair cell loss often results in a deterioration in hearing sensitivity, loss in frequency selectivity, and worse temporal precision of neural coding (Schuknecht and Gacek, 1993; Ashmore et al., 2010; Salvi et al., 2017). Moreover, atrophy of the cochlear stria vascularis was shown to occur as part of ARHL (Gates and Mills, 2005; Popelar et al., 2006).

In all studied rodent and non-human primate animal species, the synapses between IHCs and afferent auditory nerve fibers (ANFs) degenerate, due to both acoustic over-exposure and aging, before OHCs and IHCs are lost (Kujawa and Liberman, 2015; Valero et al., 2017). This cochlear synaptopathy (CS) has been shown to result in degraded neural temporal processing (Parthasarathy and Kujawa, 2018). Following the loss of cochlear synapses, primary deterioration of afferent ANFs and their spiral ganglion cells (SGCs) occurs (for a review, see Kujawa and Liberman, 2015). Some animal evidence suggests that the majority of lost ANFs are low- to medium spontaneous rate (SR) high-threshold fibers (Schmiedt et al., 1996; Furman et al., 2013), which, in humans, are thought to code moderate-to-high-level sounds, such as speech (Bharadwaj et al., 2014; Kujawa and Liberman, 2015; Huet et al., 2016). However, recent findings by Suthakar and Liberman (2021) have shown that a substantial proportion of high-SR ANFs were lost alongside low-SR ANFs in CBA/CaJ mouse following exposure to intense noise.

The extent to which lifetime noise exposure exacerbates age-related hearing difficulties has been under debate for decades and is generally poorly understood (Shone et al., 1991; Kujawa and Liberman, 2006, 2015; Ciorba et al., 2011). The majority of animal and human research has focused on how each factor separately affects cochlear hair cells and hearing thresholds, with several studies providing evidence that noise exposure may accelerate age-related threshold loss when both factors combine (Shone et al., 1991; Gates and Mills, 2005; Kujawa and Liberman, 2006; Ciorba et al., 2011; Alvarado et al., 2019; Wu et al., 2021; Fetoni et al., 2022).

Recently, consistent research efforts have been made to better understand noise-induced and age-related CS in separation using non-invasive auditory proxy measures. Animal studies have shown a clear relation between noise-induced and age-related synapse loss (occurring in separation) and objective proxy measures such as the amplitude of wave 1 of the auditory brainstem response (ABR) (Kujawa and Liberman, 2009), the middle ear muscle reflex (MEMR) threshold and amplitude (Valero et al., 2016, 2018), the envelope following response (EFR; Shaheen et al., 2015), and the ratio of the summating potential (SP) of the cochlear hair cells to the action potential (AP) of the auditory nerve (SP:AP ratio; Sergeyenko et al., 2013). A large number of human studies have investigated the effects of noise exposure and aging using objective proxy measures of CS, by employing different sample demographics, measurement techniques, and sample sizes. The findings of these studies were generally mixed and inconclusive, making it difficult to draw firm conclusions (Bramhall et al., 2017, 2019, 2021; Prendergast et al., 2017a, 2019; Valderrama et al., 2018; Carcagno and Plack, 2020, 2021; Fernandez et al., 2020).

In this narrative review paper, we will evaluate how noise exposure and aging affect peripheral auditory neural deafferentation independently using: (1) histopathological and neurophysiological; (2) electrophysiological; and (3) behavioral evidence from both animals and humans. For each type of evidence, we will discuss and compare the potential relative and combined effects between these two factors, noise exposure and aging, in relation to CS. All papers included in this review are peer-reviewed published journal articles.

## Histopathological and Neurophysiological Aspects

In this section, the histopathological and neurophysiological aspects of noise exposure, aging, and the combined effects of noise exposure and aging, will be discussed in relation to CS in both animals and humans.

### Histopathological and Neurophysiological Aspects: Noise Exposure

#### Animal Studies

Histopathological evidence from several animal species shows that acoustic over-exposure can result in significant CS in basal cochlear regions despite a near-complete recovery of hearing thresholds (Kujawa and Liberman, 2009, 2015; Lin et al., 2011; Furman et al., 2013; Maison et al., 2013; Jensen et al., 2015; Shaheen et al., 2015; Song et al., 2016; Valero et al., 2017; Hickman et al., 2018; Fernandez et al., 2020). Loss of ANFs and SGCs was noted to only be observable several months following the synapse loss in rodents (Kujawa and Liberman, 2015).

Table 1 shows a summary of key studies that investigated the proportion of synapse loss and ABR wave 1 amplitude reductions (which is a proxy measure of CS) related to noise exposure across different animal species, for which there were no permanent ABR threshold shifts. Studies suggest that different animal species exhibit variable susceptibility to noise-induced synapse loss. In these studies, the sound pressure level to which animals were exposed was selected such that it was intense enough to produce a temporary threshold shift but not result in permanent threshold elevation.

**Table 1:** Summary of key studies on the effect of noise exposure on synapse loss and ABR wave 1 amplitude across different animal species. Data reported were either explicitly mentioned in the manuscript text or were derived from the relevant figures in the respective publications using the online tool of WebPlotDigitizer version 4.5 (Rohatgi, 2021).

**Study**	**Animal species** **and gender**	**Age or weight at** **noise exposure**	**Noise exposure** **type, level, and** **duration**	**Proportion loss of synaptic** **ribbons**	**ABR Stimuli**	**Maximum ABR wave 1 reduction**
Kujawa and Liberman (2009)	Male CBA/CaJ mouse	16 weeks	Octave band of noise (8–16 kHz) at 100 dB SPL, for 2 h	Maximum of 50–60% synapse loss at basal cochlear regions	Tone pips presented at a rate of 30/s (for ABR) or 16/s (for compound action potential) at levels ranging between 10 dB SPL below the threshold to 90 dB SPL in 10-dB ascending steps	72.4% reduction at 32 kHz at 8 weeks following exposure compared to control mice using 90 dB SPL ABR stimuli
Lin et al. (2011)	Female guinea pigs (Hartley strain)	300 g	Octave band of noise (8–16 kHz) at 106- or 109-dB SPL, for 2 h	Maximum of 55% synapse loss at basal cochlear regions	Tone pips at six frequencies ranging from 2 to 32 kHz were presented at a rate of 40/s at levels ranging between 5 dB SPL below the threshold to 80 dB SPL in 5-dB ascending steps	50% reduction at 16 kHz at 2 weeks following exposure (compared to pre-exposure) using 90 dB SPL ABR stimuli
Wang and Ren (2012)	Male and female CBA/CaJ mouse	4 weeks	Octave band noise (12 kHz) at 100 dB SPL, for 2 h, for 3 exposure sessions	Maximum of 65% synapse loss; 40% synapse loss after the first and second exposure sessions. 25% additional synapse loss after the third exposure session	Tone pips or clicks were presented at a rate of 24–32 /s at levels ranging between 70- and 80-dB SPL using 5- or 10-dB ascending steps	70% reduction at 16 kHz in animals with 3 noise exposure sessions using 90 dB SPL ABR stimuli (compared to controls) 60% reduction at 16 kHz in animals with 2 noise exposure sessions using 90 dB SPL ABR stimuli (compared to controls) 40% reduction at 16 kHz in animals with one exposure session using 80 dB SPL ABR stimuli (compared to controls)
Liu et al. (2012)	Male albino guinea pigs	2–3 months (300–350 g)	Broadband noise at 105- or 110-dB SPL, for 2 h	40% synapse loss on average 1-day post-exposure: 15–35% synapse in apical regions and 60–70% synapse loss in basal regions. Synapse recovery was observed 1 month-post exposure with ribbon loss of 10% in high-frequency regions	Clicks were presented at a rate of 11.1/s at 70 dB pe-SPL	53.5% reduction at 8 kHz one month following 110 dB SPL noise exposure compared to controls 40% reduction at 4 kHz cochlear region one month following 110 dB SPL noise exposure compared to controls 24.3% reduction at 16 kHz one month following 105 dB SPL noise exposure compared to controls
Furman et al. (2013)	Female albino guinea pigs (Hartley strain)	1 month (~250 g)	Octave band noise (4–8 kHz) at 106 dB SPL, for 2 h	Maximum of 30% synapse at basal cochlear regions	Log-spaced tone pips with frequencies ranging from 2.8–45.2 Hz at a rate of 30/s and levels ranging from 10–80 dB SPL using 5-dB ascending steps	40% reduction at 16 kHz in noise-exposed animals compared to controls using 80 dB SPL ABR stimuli
Hickox and Liberman (2014)	Male CBA/CaJ mouse	16–18 weeks	Octave band of noise (8–16 kHz) at 94- or 100-dB SPL, for 2 hours	Mice exposed to 100-dB SPL had a maximum synapse loss of 44%, while those exposed to 94 dB SPL showed small non-significant synapse loss compared to controls	Tone pips of frequencies 11.3 Hz and 32 kHz presented at a rate of 40/s at a level ranging from 15–80 dB SPL in 5-dB ascending steps	36% reduction in mice exposed to 100 dB SPL noise (compared to controls) 2 weeks following exposure measured using 32 kHz ABR stimuli at 80 dB SPL 15% reduction in mice exposed to 94 dB SPL noise (compared to controls) 2 weeks following exposure measured using 32 kHz ABR stimuli at 80 dB SPL
Liberman and Liberman (2015)	Male CBA/CaJ mouse	8–9 weeks	Octave band of noise (8–16 kHz) at 98 dB SPL, for 2 hours	Maximum of 55% synapse loss at basal cochlear regions	Tone pips presented at a rate of 30/s at a level ranging from 10 dB below the hearing threshold to 90 dB SPL in 5-dB ascending steps	55% reduction in noise-exposed mice compared to controls at 45 kHz cochlear region. Wave 1 responses were averaged for ABR sound levels of 60–80 dB SPL
Möhrle et al. (2016)	Female Wistar rat	2–3 months	Broadband noise (8–16 kHz) at 100 dB SPL for 2 h	Maximum of 30% synapse loss in the mid-basal cochlear region	Clicks that cover cochlear generators ranging from 2.2 Hz to 13.8 kHz were presented at a level ranging from 20–80 dB above the threshold	35.6% reduction in young noise-exposed rats compared to controls using ABR stimuli of 65 dB above the threshold
Paquette et al. (2016)	Male and female FVB/nJ mouse	60 days post-natal (8.5 weeks)	Octave band of noise (8–16 kHz) at 105 dB SPL, for 0.5 or 1 h	Maximum of 37.5% synapse loss at basal cochlear regions	Tone pips of frequencies 8, 12, 16,24, and 32 kHz or clicks were presented at a level of 15–75 dB SPL	12% and 46% and reduction at 12 kHz 14-days following noise exposure in animals exposed to 0.5 and 1 h of noise respectively (compared to pre-noise) using 75 dB SPL ABR stimuli 69 and 75% reduction at 32 kHz 14 days following noise exposure in animals exposed to 0.5 and 1 h of noise respectively (compared to pre-noise) using 70 dB SPL ABR stimuli
Song et al. (2016)	Male and female albino guinea pig	2–3 months	Broadband noise at 105 dB SPL, for 2 h	45.1% synapse loss averaged across the cochlea at 1-day post-exposure; 17.5% synapse loss averaged across the cochlea at 1-month post-exposure	Not reported	Not reported
Valero et al. (2017)	Male and female rhesus monkey	6.5–11 years	50-Hz noise band centered at 2 kHz at 108-, 120-, 140-, and 146-dB SPL for at least 4-h one exposure session at one level	Monkeys in the temporary threshold shift group showed 12–27% synapse loss averaged across the basal half of the cochlea	Not reported	Not reported
Hickman et al. (2018)	Female chinchillas	6–9 months	Broad-spectrum (0.3–100 kHz) acoustic blast at 160–175 dB SPL, for 1.44 ms	20–45% synapse loss in mid-cochlear and basal regions	Not reported	Not reported
Fernandez et al. (2020)	Male and female CBA/CaJ mouse	16 weeks	Octave band of noise (8–16 kHz) at 97 dB SPL, for 4 h	Maximum of 50% synapse loss in basal cochlear regions	Log-spaced pips of frequencies 5.6–45.2 kHz at a level ranging from below threshold to 90 dB SPL in 5-dB ascending steps	50 and 87% reduction in mice exposed to 97 dB SPL and 100 dB SPL noise respectively 2 weeks following noise exposure at 30 kHz using ABR stimuli of 90 dB SPL

**Figure 1:**
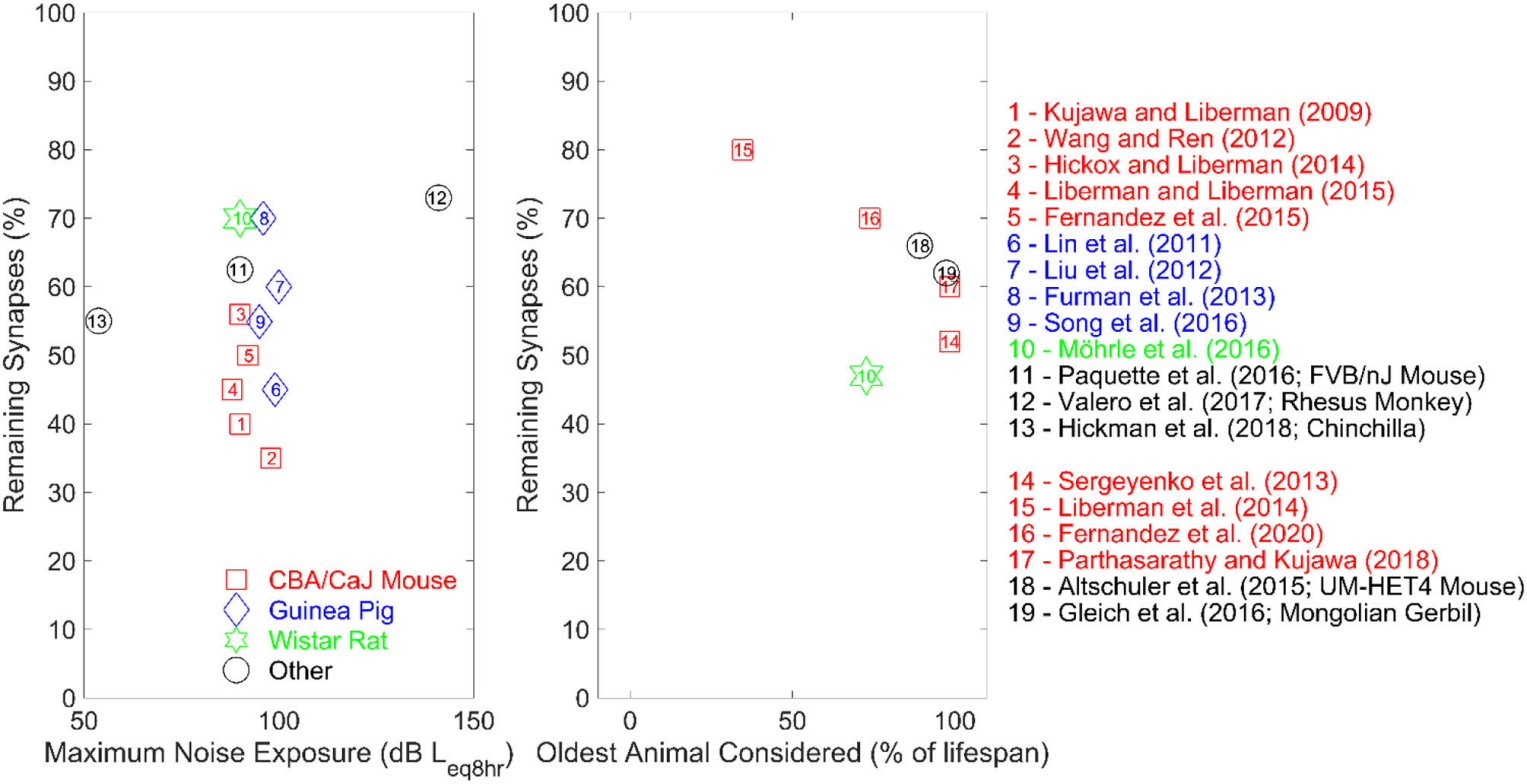
The left panel represents the proportion of remaining synapses as a function of the maximum average noise exposure of the studies summarized in Table 1. All studies exposed their subjects to octave-band noise, except for studies numbered 7, 10, and 13 employed broadband noise (study 13 only used blast noise). Studies number 2 and 12 involved multiple noise-exposure session, while all other studies exposed their subjects during one session only. The right panel shows the proportion of remaining synapses as a function of the age of the oldest animals in percent lifespan for the studies summarized in Table 2. The reference lifespan for the animals is 25 months for the Wistar rat, 36 months for the Mongolian gerbil and 30 months for both CBA and UMHET4 mouse.

As shown in Table 1, acoustic-over exposure resulted in synapse loss ranging from 12 to 70% primarily in basal regions rather than across the entire cochlea in the absence of threshold elevation in different animal species. Although the majority of the animal literature summarized in Table 1 employed octave-band noise centered at high frequencies, with few of them using broadband and blast noise insults, the differences in synapse loss could be essentially explained by the fact that the different authors investigated different types of animal species. The left panel of Figure 1 shows a scatterplot of the proportion of the remaining synapses vs. the maximum noise exposure (standardized as noise intensity in dB of equivalent continuous sound level for 8 h) considered in each study in Table 1. The different numbers, shapes, and colors of the data points in the left panel of Figure 1 reflect the different animal species that were examined in the studies in Table 1.

As inferred from the left panel of Figure 1, even for very similar noise exposure levels and durations, a wide range of synaptopathic effects were reported across the different animal species. Although animal subjects used were genetically similar in each study (which minimizes inter-subject variability due to genetic makeup), different animal species seem to exhibit different physiologic susceptibility to noise-induced CS. Interestingly, rhesus monkeys, which are physiologically closer to humans than rodents, exhibited the lowest noise-induced synapse loss compared to rodent models, which may be helpful to infer the effect of acoustic over-exposure in humans (Valero et al., 2017). Furthermore, this synapse loss in rhesus monkeys was elicited at much higher intensities than those used in rodent studies (see Figure 1), which supports the hypothesis that rhesus monkeys are less susceptible to CS. Dobie and Humes (2017) suggest that humans may be less susceptible to temporary threshold shifts following acoustic overexposure compared to rodents. These findings support the hypothesized variability in auditory system susceptibility to noise damage across different species.

Single-unit recordings suggest that the majority of ANFs lost following CS as a result of acoustic over-exposure in guinea pigs are low- and medium-SR fibers (Furman et al., 2013; Bourien et al., 2014; Song et al., 2016) which are found to represent around 40% of type I ANFs in cats and guinea pigs (Liberman, 1978; Tsuji and Liberman, 1997). In CBA/CaJ mice, significant loss of both low- and high-SR ANFs was seen following intense noise exposure (Suthakar and Liberman, 2021). Low-SR ANFs are observed to have high thresholds in several animal species such as mice, guinea pigs, cats, and gerbils; thus, they are thought to encode suprathreshold, higher-level, acoustic stimuli (Liberman, 1978; Evans and Palmer, 1980; Huet et al., 2016). However, in rhesus monkeys, Joris et al. (2011) found no evidence that low-SR fibers have higher thresholds than high-SR ANFs. This finding may therefore challenge the assumption that the loss of low-SR ANFs in humans translates into perceptual consequences at higher acoustic stimulus levels, such as SPiN difficulties (Hickox et al., 2017).

#### Human Studies

In the absence of post-mortum temporal bone data from young noise-exposed humans, it is difficult to precisely predict and quantify the extent to which CS occurs, and the noise levels, types, and duration that may produce CS before hearing thresholds are elevated. However, a recent temporal bone study by Wu et al. (2021) reported that middle-aged human subjects with a documented history of significant occupational noise exposure exhibited an additional 25% ANF loss compared to their low-noise counterparts. Moreover, OHC loss in middle-aged and older human adults with and without occupational noise exposure was highly correlated with ANF loss. Hence, the authors argued that CS may not necessarily be significant and noticeable in humans with minimal OHC loss (i.e., with normal or near-normal hearing thresholds). Instead, the effects of CS may only be clear in individuals with elevated hearing thresholds. Hence, these findings may explain the mixed and inconclusive outcomes produced by CS proxy measures in young normal-hearing humans with a history of acoustic over-exposure as discussed below.

Carney (2018) argues that although low- and medium-SR fibers may not necessarily be involved in the coding of suprathreshold stimuli in humans, their loss may still contribute to deficits in the processing of high-level acoustic stimuli through their involvement in an efferent auditory feedback loop. When this efferent feedback loop is compromised due to either noise exposure or aging, it is thought that it can no longer effectively maintain and enhance signal functional profiles at a wide range of levels and hence would not improve suprathreshold hearing in background noise (Carney, 2018).

### Histopathological and Neurophysiological Aspects: Aging

#### Animal Studies

A progressive loss of cochlear synapses and afferent ANF degeneration is observed in aging rodent models (Sergeyenko et al., 2013; Altschuler et al., 2015; Fernandez et al., 2015; Gleich et al., 2016; Möhrle et al., 2016; Parthasarathy and Kujawa, 2018; Peineau et al., 2021). Table 2 shows a summary of key animal studies which investigated the proportion of synapse loss and the reduction in the amplitude of wave 1 of the ABR in relation to aging across different rodent species. The right panel of Figure 1 shows a scatterplot of the proportion of remaining synapses as a function of the age of the oldest age of animals (in percent lifespan) considered in the studies summarized in Table 2. The different numbers, shapes, and colors of the data points in the right panel of Figure 1 reflect the different animal species that were examined in the studies in Table 2.

**Table 2:** Summary of the key studies on the effect of aging on synapse loss and ABR wave 1 amplitude across different animal species. Data reported were either explicitly mentioned in the manuscript text or were derived from the relevant figures in the respective publications using the online tool of WebPlotDigitizer version 4.5 (Rohatgi, 2021).

**Study**	**Animal** **species/gender**	**Age of animals**	**Percentage loss of synaptic ribbons**	**ABR stimuli**	**Maximum percentage of the ABR wave 1** **reduction**
Sergeyenko et al. (2013)	Male CBA/CaJ mouse	4–144 weeks	Maximum of 48% synapse loss at 144 weeks compared to 4 weeks. Age-related synapse loss was fairly uniform across all cochlear regions Maximum of 40% synapse loss at 128 weeks compared to 4 weeks. Age-related synapse loss was fairly uniform across all cochlear regions	Log-spaced tone bursts with frequencies 5.6–45.2 kHz presented at a level ranging from below 5 dB below the threshold to 90 dB SPL in 5-dB ascending steps	95% reduction in 128-week mice compared to 4-week mice at 12 kHz measured using 80 dB SPL ABR stimuli 80% reduction in 96-week mice compared to 4-week mice at 12 kHz measured using 80 dB SPL ABR stimuli 71.5% reduction in 80-week mice compared to 4-week mice at 12 kHz measured using 80 dB SPL ABR stimuli
Liberman et al. (2014)	Male CBA/CaJ mouse	6–45 weeks	Synapse loss in age controls at 45 weeks ranged between 2–20% depending on cochlear location. The proportion of synapse loss in apical and basal areas seems similar (about 10–20%)	Tone busts presented at a rate of 35/s and with a level ranging from 5 dB below the threshold to 80 dB SPL ascending in 5-dB steps	35% in 45-week age-only control mice compared to 8-week control subjects at 17 kHz. Responses were averaged for ABR stimuli ranging between 60–80 dB SPL
Altschuler et al. (2015)	Female UM-HET4 mouse	Three groups: 5–7, 22–24, and 27–29 months	The two older groups exhibited 20–34% synapse loss compared to the young group averaged across cochlear regions examined (i.e., 1–4 mm from the apex). Synapse reduction was significantly less in the 22–24-month group compared to the 5–7-month group. No further significant synapse loss was noted in the 27–29-month group compared to the 22–24-month group in all synapse regions studied	Not reported	Not reported
Fernandez et al. (2015)	Male CBA/CaJ mouse	16–104 weeks	Up to 30% synapse loss in 22.6 kHz cochlear region in age-only controls 96 weeks following noise exposure compared to young controls at 4 weeks following noise exposure. The proportion of age-related synapse loss ranged between 15–30% across different cochlear regions in older age-only controls at 96-weeks following noise exposure	Log-spaced tone bursts of frequencies ranging between 5.6–45.2 kHz were presented at a rate of 30/s at a level from 30–90 dB SPL ascending in 5-dB step increments	66% in 88 weeks following noise exposure (at the age of 104 weeks) in age-only older controls compared to 2 weeks following noise exposure (at the age of 18 weeks) in young controls at 32 kHz using 90 dB SPL ABR stimuli
Gleich et al. (2016)	Mongolian gerbil	Two groups: about 10 and about 38 months	The older group exhibited 21% synapse loss on average (across the entire cochlea) and a maximum of 38% loss at apical cochlear regions compared to the younger group	Not reported	Not reported
Möhrle et al. (2016)	Female Wistar rat	Three pre-noise exposure groups: 2–3, 6–10, and 19–22 months.	The pre-noise exposure groups aged 19–22 months and 6–10 months exhibited 53 and 29% synapse loss respectively in mid-basal cochlear regions compared to the 2–3-month group (pre-noise exposure)	Clicks that cover cochlear generators ranging from 2.2 Hz to 13.8 kHz were presented at a level ranging from 20–80 dB above the threshold	The pre-noise exposure groups of 19–22-months and 6–10-months both exhibited a reduction in the ABR wave 1 amplitude of 40 and 35.6% respectively compared to the 2–3-month pre-noise exposure group at 75 dB above threshold ABR stimuli
Parthasarathy and Kujawa (2018)	Male and female CBA/CaJ mouse	16–128 weeks	Maximum of 40% synapse loss by 128 weeks. A fairly similar age-related pattern of synapse loss in mid-basal and basal cochlear regions	Log-spaced tone bursts ranging from 5.6–45.2 kHz were presented at a rate of 33/s at levels ranging from 10–90 dB SPL	84, 71.1, 50, and 23.4% in 128, 108, 64, and 32- weeks mice respectively compared to 16-week mice at 32 kHz using 90 dB SPL ABR stimuli 84.5, 69, 39.4, and 29.9% in 128, 108, 64, and 32-weeks mice respectively compared to 16-week mice at 12 kHz using 90 dB SPL ABR stimuli

Unlike acute noise-induced CS, which primarily manifests in basal cochlear regions, Fernandez et al. (2015) provided evidence that the cochlear region of noise-induced CS broadens over time to have a widespread impact after a single acoustic trauma. Moreover, age-related synapse loss did not exceed 50% across the different rodent species, whereas acoustic over-exposure seems to account for a higher proportion of synapse loss in some animal studies (Kujawa and Liberman, 2009; Lin et al., 2011; Liu et al., 2012; Singer et al., 2013; Liberman and Liberman, 2015). Furthermore, unlike noise-exposure studies, evidence from aging studies suggests progressive age-related OHC loss that occurs in parallel with synapse loss. A minimal loss of IHCs took place as age progressed and SGC deterioration was slow and uniform across the different cochlear regions (Sergeyenko et al., 2013; Parthasarathy and Kujawa, 2018). Similar to noise-induced CS, the ANFs lost as a result of aging are thought to be predominantly low- to medium-SR fibers (Schmiedt et al., 1996; Kujawa and Liberman, 2015).

#### Human Studies

Post-mortem human temporal bone studies have confirmed a significant age-related degeneration of SGCs (Otte et al., 1978; Kusunoki et al., 2004; Makary et al., 2011; Nayagam et al., 2011). The percentage of SGC loss seems to be greater in humans with a higher proportion of degenerated cochlear hair cells. For instance, Makary et al. (2011) estimated the rate of SGC loss at around 1,000 per decade in human subjects with normal counts of cochlear hair cells. Otte et al. (1978) reported that this SGC loss rate was doubled (i.e. around 2,000 per decade) in subjects with varying degrees of sensorineural hearing loss compared to subjects with normal cochlear hair cells as shown in the data of Makary et al. (2011). The process of aging seems to affect type I ANFs in humans (Felder and Schrott-fischer, 1995; Chen et al., 2006) such that older adults with high-frequency sensorineural hearing loss were found to exhibit 30–40% type I ANF neuronal degeneration in the absence of significant IHC or SGC loss (Felder and Schrott-fischer, 1995).

More recently, Wu et al. (2019) found that the degeneration of type I ANF peripheral axons due to aging in humans took place well before the loss of OHCs, IHCs, and SGCs. Hence, this is consistent with the primary nature of age-related ANF deafferentation in humans. More than 60% ANF loss (as averaged across the entire standard audiometric range) was estimated to have occurred in human subjects aged over 50 years (Wu et al., 2019). ANF deafferentation was hypothesized to result in the loss of auditory neural information channels, which may render it more difficult for older adults to centrally process speech in the presence of background noise, even when hearing thresholds are within normal limits (as reflected by the normal counts of OHCs) (Wu et al., 2019). However, a caveat to this assumption could be that the relative proportion of low- to medium SR fibers, and their role in higher-level speech perception, are poorly understood in humans.

Wu et al. (2021) determined ANF loss in post-mortum human temporal bones of subjects aged 43–104. The authors estimated age-related ANF loss at 6.3% per decade. This was noted to take place across the entire human cochlea with more pronounced effects in basal cochlear regions. However, unlike the data reported by Wu et al. (2019), Wu et al. (2021) showed a strong positive correlation between OHC and ANF loss. According to the authors, this positive correlation between OHC and ANF loss contradicts the hypothesized primary nature of ANF loss in humans and hence adds more uncertainty to how age-related CS manifests perceptually in humans with normal/near-normal audiometric profiles. This is because most ANFs that are affected by CS are thought to make contact with IHCs and histopathological animal studies have demonstrated that the loss of CS and afferent ANFs occurs well before OHCs are lost (as discussed earlier). More temporal bone evidence is therefore necessary to establish the relation between ANF and OHC loss over the entire human lifespan.

Viana et al. (2015) counted synaptic ribbons connected with IHCs in older humans and reported that aged ears had no more than 2.0 synapses per IHC at basal cochlear regions (i.e., at about 2 kHz) compared to 11.3–13.3 synapses per IHC in young controls. This translates to approximately 85% age-related basal synapse loss in humans. At more apical cochlear regions (e.g., 0.25 kHz), synapses per IHC did not exceed 7.6 in older ears (i.e., about 40% synapse loss), which suggests that age-related synapse loss in humans may have a bigger impact at basal rather than apical cochlear regions. Synapse loss was reported to take place well before cochlear hair cells were lost. This is thus consistent with Wu et al. (2019) findings concerning the primary nature of peripheral neural deafferntiation. Bharadwaj et al. (2014) predicted that age-related synapse loss most likely occurs at a minimum of 30% in aged humans. This prediction was inferred from mouse data which showed that SGC degeneration occurred 1–2 years following synapse loss. Moreover, this prediction is consistent with the findings of Viana et al. (2015) and with rodent studies summarized in Table 2 which documented age-related synapse loss of up to 50%. Hence, significant synapse loss may well occur over a human's lifespan given the existing evidence from temporal bones on age-related ANF and SGC degeneration in older humans.

### Histopathological and Neurophysiological Aspects: Combined Effects of Noise Exposure and Aging

#### Animal Studies

In a few animal models, the combined impact of aging and noise exposure on synapse loss has been investigated. Fernandez et al. (2015) determined the pattern of auditory neural degeneration following acute noise exposure across the lifespan of CBA/CaJ mice. Synapse loss was estimated at a maximum of about 55% in older animals aged 96 weeks following exposure to 100 dB SPL noise for 2 h at the age of 16 weeks compared to up to 30% in non-exposed older counterparts. Synapse loss was most significant in basal cochlear regions in both young and older mice. As noise-exposed mice aged further, synapse counts in more apical cochlear regions were found to deteriorate as well. The authors noted, however, that cochlear regions with the most significant noise-induced synapse loss exhibited less synapse degeneration per year (throughout the 96 weeks following the noise exposure) compared to cochlear areas with the lowest noise-induced CS. The authors proposed that this decrease in synapse loss is consistent with the assumption that only a proportion of afferent auditory ANFs may be vulnerable to both noise exposure and aging (Schmiedt et al., 1996; Furman et al., 2013).

Möhrle et al. (2016) reported that young rats exposed to 100 dB SPL noise for 2 h exhibited about 30% synaptic loss in mid-basal cochlear regions compared to controls. The synapse populations following the same noise exposure event in middle-aged and old rats were not significantly different from controls in each age group. Moreover, synaptic counts in middle-aged noise-exposed rats were similar to young noise-exposed animals. Old noise-exposed rats had about 15% fewer mid-basal IHC-ANF synapses compared to their young noise-exposed counterparts.

#### Human Studies

**Figure 2:**
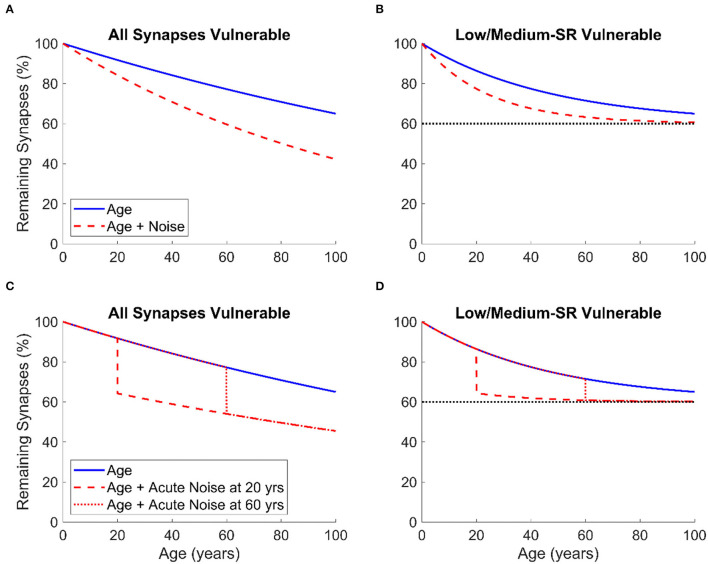
The proportion of remaining IHC-ANF synapses at basal cochlear regions as a function of age in humans given two models of synapse/ANF vulnerability: All synapses vulnerable **(A,C)** and only low- and medium-SR ANFs vulnerable **(B,D)**. The two models are based on two assumptions: regular constant lifetime acoustic over-exposure **(A,B)** and one single event of intense noise exposure occurring at age 20 or 60 **(C,D)**. In **(B,D)**, the dashed line is an asymptotic line defining the percentage of synapse loss beyond which no further CS occurs.

By assuming either a regular constant acoustic over-exposure throughout the lifespan or exposure to one single event of intense noise, we propose two simple models for the combined effects of noise exposure and aging on CS in basal cochlear regions as shown in Figure 2. In this figure, the proportion of remaining synapses is expressed as a function of age ranging from 0 to 100 years. Figures 2A,B represent the effects of age and the combined effects of age and constant acoustic overexposure on the proportion of synapse loss, while Figures 2C,D illustrate the effects of age and the combined effects of age and a single event of intense noise exposure. For both instances of noise exposure scenarios, we assume that either all IHC-ANF synapses (Figures 2A,C) or only low- and medium-SR ANFs (Figures 2B,D), which are thought to comprise 40% of type I ANFs in cats and guinea pigs (Liberman, 1978; Tsuji and Liberman, 1997), are vulnerable. It is assumed in the models that age causes the loss of a constant proportion of the remaining vulnerable synapses per unit of time. Similarly, noise exposure is assumed to cause a constant proportional loss of the remaining vulnerable synapses (for a given exposure). In other words, for a given vulnerable synapse, there is assumed to be a constant risk of loss for a given unit of time, or a given exposure. This is why the plots are asymptotic curves, rather than straight lines.

For both noise exposure scenarios of our models, we predict that, although human temporal bone studies have shown that age-related ANF loss may occur at a proportion of more than 60% (Wu et al., 2019), IHC-ANF synapse loss secondary to aging may take place at a more conservative proportion (i.e., 30% in basal cochlear regions) as suggested by Bharadwaj et al. (2014). It is important to acknowledge that the main limitation in temporal bone studies, which may reduce confidence in their findings, is that many human subjects were in poor health prior to death. This may result in over-estimating the effects of aging (since there may be factors other than age contributing to CS and the influence of these factors may increase with age). Moreover, these studies lack precise estimation of noise and ototoxic exposure. Individuals who were not identified as having an occupational noise history could still have had significant lifetime exposure to noise and/or ototoxins. Finally, this difference in ratios may be explained by factors other than synapse loss that may account for ANF degeneration such as age-related genetic susceptibility to ANF degeneration.

We also assume that about 30% further synapse loss occurs due to acoustic over-exposure for both noise exposure scenarios. This estimation is based on Valero et al. (2017) data which has shown that 12–27% synapse loss occurred in the non-human primates of macaque monkeys following one intense event of noise exposure. Unfortunately, no animal or human data are available on the proportion of synapse loss secondary to cumulative regular constant lifetime noise exposure. So, we arbitrarily extended the assumption of 30% synapse loss to the scenario of regular acoustic-over exposure across the entire human lifespan.

For the assumption in which all synapses are vulnerable and for both scenarios of noise exposure (Figures 2A,C), CS due to noise exposure has a greater overall effect as more synapses are vulnerable. In contrast, synapse loss, either due to aging only or to noise exposure and aging together, saturates to a maximum of 40% if only low- and medium-SR ANFs are vulnerable (assuming that humans have the same proportion of low- and medium-SR ANFs to cats and guinea pigs as discussed above) as shown in Figures 2B,D.

It is worth pointing out that this model (as proposed in Figure 2) is very simplistic and is intended to be primarily a schematic illustration of the patterns of synapse loss that may occur in human ears secondary to noise exposure throughout the lifespan. However, the model may be useful for relating the expected consequences of different combinations of noise exposure and aging to objective and behavioral proxy measures in animals and humans.

Recently, the combined impact of both occupational noise exposure and aging in post-mortum human temporal bones was assessed by Wu et al. (2021). Lifetime occupational noise exposure was found to uniformly exacerbate age-related ANF loss across the different cochlear regions in the middle-aged group (i.e., subjects aged 50–74) by 25%, but not in the older group (i.e., subjects aged 75–104). These results are broadly consistent with the assumption we made above that when only low- and medium SR ANFs are vulnerable to both noise exposure and aging, little further CS occurs at older ages once a specific proportion of IHC-ANF synapses has been lost (Figures 2B,D). It is important to point out, however, that for the highest cochlear frequency regions considered by Wu et al. (2021) almost all ANFs were lost where a near-complete degeneration of IHCs had occurred. Therefore, the primary cause of this high-frequency ANF loss may not necessarily be CS, but rather IHC loss. This is because the loss of an IHC will lead to degeneration of the associated ANFs, irrespective of the degree of CS.

Wu et al. (2021) reported that IHC loss due to occupational noise exposure was minimal. In contrast, a high correlation between ANF and OHC loss in both basal and apical cochlear regions across different subjects of varying ages and with and without documented occupational noise exposure was found. Hence, the authors suggest that the effects of CS may only be substantial in the presence of threshold elevation in humans. Furthermore, OHC loss, rather than IHC or ANF loss, was found to be the main predictor of subjects' word recognition in quiet.

Given the lack of human temporal bone studies on the effect of noise exposure in isolation, it is difficult to estimate precisely how a history of acoustic over-exposure may impact the populations of cochlear synapses and ANFs at an older age. Given the difficulty in planning and conducting temporal bone studies, it is likely some time before data are available on how noise exposure and aging interact. This lack of studies may stem in part from the fact that it is difficult to retrospectively quantify the extent of lifetime noise exposure in deceased humans. Moreover, such studies may not be successful in controlling for genetic factors and past exposure to ototoxic substances, which may influence the onset and progression of age-related cochlear degeneration as well as the vulnerability to noise exposure at both young and older ages (Pyykkö et al., 2007).

## Objective Proxy Measures of Cochlear Synaptopathy

In this section, animal and human studies in relation to noise exposure, aging, and the combined effects of noise exposure and aging, will be discussed in the framework of the objective proxy measures of CS: ABR wave I, ABR wave I:V amplitude ratio, SP:AP ratio, EFR, and MEMR.

### Auditory Brainstem Response Wave I

#### Auditory Brainstem Response Wave I: Noise Exposure

##### Animal Studies

Across different animal species, noise-induced CS, primarily in the absence of hair cell loss, is associated with a 12–72.4% decrease in the amplitude of wave 1 of the ABR to moderate-high level stimuli, as summarized in Table 1. In addition to the fact that these studies involved different animal species (which likely exhibit different susceptibility to noise-induced CS), different studies used an exposure of different levels, durations, and spectra of noise. Moreover, the effect of noise exposure was investigated using different ABR stimuli, and measures were made at different frequencies (which may be affected by CS to differing extents). These methodological differences, highlighted in Table 1, could at least partially explain the high variability in the percentage of the ABR wave 1 reduction found across the different animal studies. Finally, since the majority of the animal literature summarized in Table 1 employed animals of single-sex, it is difficult to draw firm conclusions on whether the amplitude of ABR wave 1 varies, and to what extent, as a function of sex.

##### Human Studies

The effect of excessive noise exposure on the amplitude of wave I of young normal-hearing human adults has been inconclusive. Some studies have reported that a smaller amplitude of wave I of the ABR is associated with high noise exposure in young subjects (Stamper and Johnson, 2015a,b; Liberman et al., 2016; Bramhall et al., 2017, 2021; Valderrama et al., 2018; Buran et al., 2022), while several other studies failed to document such an effect (Grinn et al., 2017; Grose et al., 2017; Prendergast et al., 2017a, 2018; Skoe and Tufts, 2018; Couth et al., 2020). Table 3 shows a summary of studies that investigated the effect of noise exposure on ABR wave I amplitude in humans. It is worth highlighting that Bramhall et al. (2017, 2021) investigated firearm exposure among military veterans, which is primarily an impulsive type of noise and may hence be different in effect from the recreational exposures considered by the majority of the other human literature (for reviews, see Bramhall et al., 2019; Le Prell, 2019). As highlighted in Table 3, the amplitude of ABR wave I of female participants was larger than that of males (Stamper and Johnson, 2015a,b; Bramhall et al., 2017; Grose et al., 2017; Prendergast et al., 2017a; Valderrama et al., 2018). ABR wave amplitudes seem to be influenced by the sex of participants due to the potential variability in lifetime noise exposure (i.e., males may exhibit higher noise exposure than females; Stamper and Johnson, 2015b), and anatomical differences between sexes (such as differences in cochlear dispersion, head size, and bone density; Mitchell et al., 1989; Don et al., 1993). The influence of sex on ABR wave I was not quantified and controlled in all human CS studies. Future studies on CS in humans could be more explicit in considering this factor.

**Table 3:** Summary of the methods and findings of the studies that investigated the effect of noise exposure on the amplitude of wave I of the ABR in humans. Data reported were either explicitly mentioned in the manuscript text or were derived from the relevant figures in the respective publications using the online tool of WebPlotDigitizer version 4.5 (Rohatgi, 2021).

**Study**	**Participants**	**ABR recording parameters**	**Outcomes**	**Sex-specific findings**
Stamper and Johnson (2015a,b)	30 subjects (20 females). Age 18–29 years. All had normal hearing (hearing thresholds <20 dB HL at 0.25–8 kHz). Participants had various amounts of self-reported lifetime noise exposure. Participants with high lifetime noise exposure were recruited from University music departments	Mastoid and tympanic membrane electrode montages. Click and tone burst at 4 kHz were used at the level of 90 dB nHL and subsequently lowered by 10 dB steps	In Stamper and Johnson (2015a), the ABR wave I amplitude was 42.7% (*p* = 0.015) and 35.4% (*p* = 0.095) smaller on average in high noise subjects compared to low noise counterparts measured using clicks at 90 dB nHL with mastoid and tympanic membrane electrode montages respectively. Measurements using tone bursts of 4 kHz at 90 dB nHL showed the ABR wave I amplitude reduction at 48% (*p* = 0.013) and 43.3% (*p* = 0.056) on average in high noise subjects using mastoid and tympanic membrane electrode montages respectively.	Sex was a confound, with males having the highest noise exposures and the lowest wave I amplitudes Stamper and Johnson (2015a) In a reanalysis, Stamper and Johnson (2015b) reported that the ABR wave I amplitude reductions measured using clicks at 90 dB nHL were only statistically significant (in females (*p* = 0.005), not males (*p* = 0.302; i.e., 43.3% lower wave I amplitudes in high noise females compared to low noise females)
Liberman et al. (2016)	34 young adults (15 females) aged 18–41 were recruited from local colleges and universities in the USA. Participants were allocated into high-risk (*n* = 22) and low-risk (*n* = 12) for ear damage based on self-reported noise exposure	94.5 dB nHL clicks at a rate of 9.1 Hz or 40.1 Hz. In order to eliminate the contribution of the contralateral ear, ipsilateral clicks were presented with a contralateral broadband masker at 55 dB nHL. Ipsi- and contra-lateral tiptroad ear canal montage was used	The high-risk group had a 14.7% smaller ABR wave I amplitude compared to the low-risk group (*p* < 0.001).	The authors repeated the analyses across both sexes of participants separately in order to evaluate any sex effect. The differences originally found remained highly significant in both sex groups after the analyses were run on male- and female-only groups
Bramhall et al. (2017)	100 military veterans and nonveterans aged between 19–35 years. Participants were divided into four groups based on self-reported noise exposure: non-veterans, non-veteran firearm, veteran high noise, and veteran low noise. All participants had normal hearing thresholds	Tone bursts at 1, 3, 4, and 6 kHz at levels ranging between 60 and 110 dB p-peSPL using extra-tympanic electrodes	Measurements obtained at 110 dB p-peSPL: - Using a 1 kHz tone burst ABR wave I amplitude was 33.3% smaller in non-veteran firearm compared to non-veterans and 53.3% smaller in veteran high noise compared to veteran low noise. - Using a 3 kHz tone burst, the ABR wave I amplitude was 22.6 and 33.3% smaller in non-veteran firearm compared to non-veterans and in veteran high noise compared to veteran low noise respectively - Using a 4 kHz tone burst, the ABR wave I amplitude was 20.5 and 26.2% smaller in non-veteran firearm compared to non-veterans and in veteran high noise compared to veteran low noise respectively - Using a 6 kHz tone burst, the ABR wave I amplitude was 15.6 and 16.7% smaller in non-veteran firearm compared to non-veterans and in veteran high noise compared to veteran low noise respectively	A weak sex effect was seen such that females had greater wave I amplitude than males in the veteran high-noise group and the non-veteran group. The ABR wave I sex differences were smaller than the mean ABR wave I differences (across both sexes) between the veteran high-noise and non-veteran groups. Males had slightly smaller wave I amplitudes than females in veteran high-noise and non-veteran groups using different tone burst intensities at 4 kHz
Grinn et al. (2017)	32 participants (19 females) aged between 21–27 years with normal hearing as defined by hearing thresholds of ≤ 25 dB HL at 0.25–8 kHz	Clicks and tone bursts at 2, 3, and 4 kHz were presented at a level of 70 dB HL, 80 dB HL, and 90 dB HL at a rate of 11.7/s. In-the-canal tiptrode electrode configuration was used with non-inverting and ground electrodes stacked with spacing at midline high forehead (Fz)	After controlling for sex, noise exposure did not predict ABR wave I amplitudes using clicks (*p* = 0.25; for males r = 0.0736, *p* = 0.82; for females r = −0.0754, *p* = 0.759) and tone bursts at 2 kHz (*p* = 0.88; for males r = −0.114, *p* = 0.724; for females r = −0.0791, *p* = 0.747), 3 kHz (*p* = 0.71; for males r = 0.0346, *p* = 0.915; for females r = −0.0634, *p* = 0.803), and 4 kHz (*p* = 0.22, for males r = −0.008, *p* = 0.98; for females r = −0.129, *p* = 0.598) at 90 dB nHL	Females had significantly larger wave I amplitudes than males at 90 dB HL (for clicks *p* = 0.002; for 2 kHz *p* = 0.006; for 3 kHz *p* = 0.004; for 4 kHz *p* < 0.001)
Prendergast et al. (2017a)	126 participants (75 females) aged between 18–37 years with normal hearing thresholds ( ≤ 20 dB HL at 0.5–8 kHz)	Band-pass filtered clicks with a bandwidth from 1.5–4 kHz were presented at 80- and 100- dB peSPL at a rate of 11 clicks/s. Active electrodes were placed at the high forehead (Fz), the seventh cervical vertebra (C7), and the left and right mastoids (M1)	Noise exposure did not predict ABR wave I amplitudes at 80 dB peSPL (r = −0.07, *p >* 0.05) and 100 dB peSPL levels (r = −0.1, *p >* 0.05)	Females had larger ABR wave I amplitudes than males at 100 dB peSPL
Grose et al. (2017)	61 participants (29 females) aged between 18–35 with normal hearing as defined by hearing thresholds of ≤ 20 dB HL at 0.25–8 kHz. Participants were divided into two groups: the experimental group (n=31; had exposure to recreational noise) and the control group (*n* = 30; minimal exposure to recreational noise)	Clicks were presented at 95- and 105- dB ppeSPL at a rate of 7.7 clicks/s. An electrode montage of the ear-canal electrode (Tiptrode) as the inverting electrode was used for the test ear; the non-inverting electrode was placed midline on the high forehead and the ground electrode between the eyebrows	For both 95- and 105- dB ppeSPL presentation levels, the experimental group had lower ABR wave I amplitudes compared to the control group, however, the differences in ABR wave I amplitudes across both groups were not statistically significant (*p* = 0.67)	Males had significantly smaller ABR wave I amplitudes in both groups compared to females
Prendergast et al. (2018)	30 female participants aged 19–34 with normal hearing as defined by hearing thresholds of ≤ 20 dB HL at 0.25–8 kHz. Participants were divided equally into two groups based on lifetime noise exposure: the low-noise group (*n* = 15) and the high-noise group (*n* = 15)	Band-pass filtered clicks with a bandwidth of 0.1–1.5 kHz were presented at 80 dB nHL at a rate of 11 clicks/s. Two different electrode montages were used: mastoid electrode and canal tiptrode	Although the low-noise group had smaller ABR wave I amplitudes across both electrode montages compared to the high-noise group, the differences in ABR wave I amplitudes were not statistically significant (*p* > 0.05)	Not applicable
Valderrama et al. (2018)	74 participants (37 females) aged between 29–55 years. 84% of participants had normal hearing thresholds defined as ≤ 20 dB HL from 0.25–6 kHz	108.5 peSPL clicks using two reference electrode montage setups: ipsilateral mastoid (Fz-Tp9/Tp10) and ipsilateral ear canal (Fz-TIP)	After controlling for sex, the amplitudes of waves I, III, and V of ABR were smaller by 43.1, 60.7, and 45.4% respectively for participants with the 10% highest lifetime noise exposure units using Fz-Tp9/Tp10 electrode configuration compared to subjects with the lowest 10% lifetime noise exposure units. After controlling for sex and using the Fz-TIP electrode configuration, the amplitudes of waves I, III, and V of the ABR were smaller by 43.4, 63.7, and 41.1% respectively for participants with 10% highest lifetime noise exposure units compared to those with the lowest 10% lifetime noise exposure units Given all participants with various noise exposures, noise exposure was a significant predictor of ABR wave I amplitudes using Fz-Tp9/Tp10 montage (*p* = 0.0038) and Fz-TIP montage (*p* = 0.0215)	Males exhibited smaller ABR wave I amplitude compared to females
Skoe and Tufts (2018)	55 participants (41 females) aged between 18–24 years were divided into two groups based on lifetime noise exposure: the low-exposure group (n = 29) and the high-exposure group (n = 26). All participants had normal hearing thresholds defined as ≤ 25 dB HL from 0.25–8 kHz	Clicks were presented at 75 dB nHL at eight presentation rates of 3.4, 6.9, 10.9, 15.4, 31.25, 46.5, 61.5, and 91.24 clicks/s. The non-inverting electrode was placed on the central vertex of the head (Cz), the inverting electrode was placed on the right earlobe (A2), and the ground electrode was placed on the forehead	No statistically significant difference in ABR wave I amplitude across different click rates between the low-exposure and high-exposure groups for either the peak-to-baseline wave I measure (*p* = 0.73) or the peak-to-trough wave I measure (*p* = 0.88). However, there was a trend of slightly smaller ABR wave I amplitudes for the high-noise exposure group compared to the low-exposure group across all click rates except for the 91.24 clicks/s	No statistically significant difference in ABR wave I between males and females across both the peak-to-baseline wave I measure and the peak-to-trough wave I measure. However, females had a trend of higher ABR wave I amplitudes compared to males in the peak-to-trough wave I measure, but not in the peak-to-baseline wave I measure
Couth et al. (2020)	137 participants (66 females) aged between 18–27 years. Participants were divided into two groups: musicians (n = 76) and non-musicians (n = 47). All participants had normal hearing thresholds defined as ≤ 20 dB HL from 0.25–8 kHz except for 4 participants who had mild hearing loss (hearing thresholds between 25–40 dB HL)	Clicks were presented at a level of 60 dB HL and 80 dB HL using a click rate of 11.1/s. A single-channel vertical montage configuration was used with the active electrode placed at Fz (high forehead), the reference electrode on the ipsilateral mastoid, and the ground electrode on the contralateral mastoid	Both musicians and non-musicians with high noise exposure exhibited statistically similar ABR wave I amplitudes (*p >* 0.05) compared to low-noise musicians and non-musicians respectively using both 60 and 80 dB nHL stimuli. There was a trend of non-significantly smaller ABR wave I amplitudes across high noise participants in both the musician and non-musician groups compared to their low-noise counterparts in both groups using the 60 dB nHL stimulus level	The authors did not control for the sex of participants in the analyses of ABR wave I amplitudes
Bramhall et al. (2021)	79 young audiometrically-normal participants (defined as having hearing thresholds of ≤ 20 dB HL from 0.25–8 kHz) aged 19–35 were divided into 3 groups: military veteran high noise (*n* = 30, 6 females), military veteran medium noise (*n* = 18, 10 females), and non-veteran control (*n* = 31, 17 females)	4 kHz tone bursts were presented at 90, 100, and 110 dB peSPL and a rate of 11.1/s. Ipsilateral ear canal montage was used	The posterior probability that the mean ABR wave I amplitude is greater for non-veteran controls than for high noise veterans at stimulus levels of 90, 100, and 110 dB pe- SPL was 94, 71, and 51%, respectively	No sex-specific noise exposure effects on ABR wave I amplitudes were found in all subgroups

#### Auditory Brainstem Response Wave I: Aging

##### Animal Studies

Rodent studies suggest that age-related CS, in the absence of significant lifetime noise exposure, results in reduced amplitude of wave 1 of the ABR as documented in Table 2. The maximum age-related decline in wave 1 amplitude ranged between 70 and 90% (Sergeyenko et al., 2013; Parthasarathy and Kujawa, 2018), which is generally greater than that seen in studies investigating the effect of noise exposure in young animals (summarized in Table 1). This difference could be explained by the fact that age-related OHC loss had occurred in older animal subjects (which was not the case in young noise-exposed animals) especially in basal cochlear regions as documented by studies such as Sergeyenko et al. (2013), Liberman et al. (2014), Fernandez et al. (2015), and Parthasarathy and Kujawa (2018). Moreover, it is possible that aging and noise exposure result in different degrees of synapse and ANF loss depending on cochlear location and spontaneous rate level.

Since the ABR wave 1 amplitudes evoked by frequency-specific tone bursts are highly dependent on basal cochlear generators, as data from guinea pigs have shown (Eggermont, 1976), age-related basal OHC loss may further decrease the magnitude of the ABR wave 1 and thus obscure the effect caused by CS. It is worth pointing out that the ABR wave 1 amplitude reductions were seen to take place across all stimulation frequencies (i.e., low- and high-frequency tone bursts) in the animal studies summarized in Table 2. Based on this assumption, the pure effect of CS on the ABR wave 1 amplitude evoked by frequency-specific tone bursts can therefore only be determined once age-related basal OHC loss has been controlled for. However, computational modeling data from Verhulst et al. (2018a) suggest that OHC loss may have a limited impact on ABR wave 1 amplitudes for stimuli of 90 dB peSPL since the response growth of the OHCs is linear at high stimulus intensities. The computational modeling found that OHC loss even slightly increased ABR wave 1 amplitude for stimulus levels above 90 dB peSPL (Verhulst et al., 2018a). Moreover, Buran et al. (2022) also showed that accounting for cochlear gain loss (based on pure tone thresholds or distortion product otoacoustic emissions) in a computational modeling algorithm had a small effect on synapse predictions generated by the model from the ABR wave I amplitude measurements.

**Figure 3:**
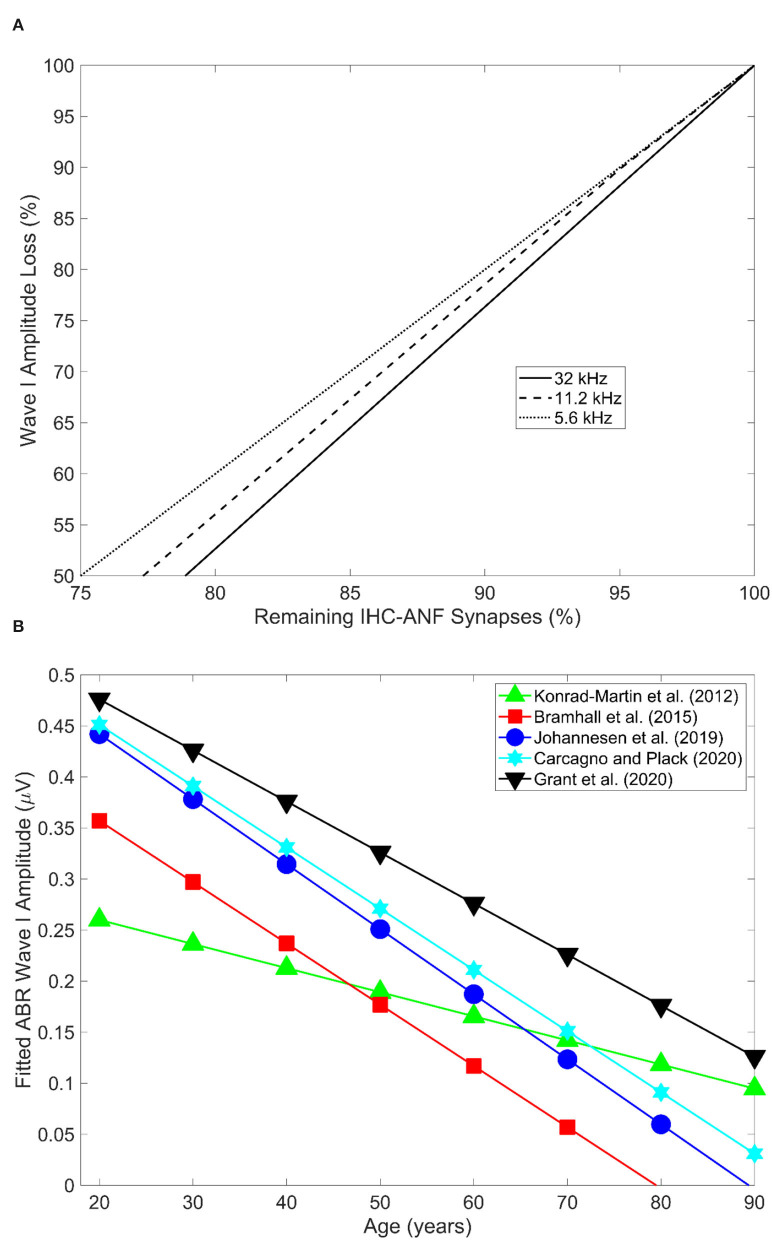
**(A)** Shows the relation between age-related decline in wave 1 amplitude and remaining IHC-ANF synapses as estimated in the 5.6, 11.2, and 32 kHz cochlear regions in CBA/CaJ mice. Redrawn from the data reported in panel D of Figure 5 in Sergeyenko et al. (2013) using the online tool of WebPlotDigitizer version 4.5 (Rohatgi, 2021). **(B)** Illustrates ABR wave I amplitude as a function of age across five different human studies. Redrawn from the data reported in Figure 4 in Bramhall (2021) using the online tool of WebPlotDigitizer version 4.5 (Rohatgi, 2021).

A strong correlation has been reported between the proportion of age-related synapse loss and ABR wave 1 amplitude in mice (Sergeyenko et al., 2013; Parthasarathy and Kujawa, 2018). Figure 3A illustrates the relationship from the results of Sergeyenko et al. (2013). It is important to point out that in this correlation analysis age-related OHC loss was never accounted for, and thus, the reductions in the ABR wave 1 amplitudes could be confounded by age-related threshold shifts. Further research is necessary to establish the effect of OHC loss on ABR wave 1 amplitude reduction secondary to CS (for the reasons discussed above) in order to establish whether ABR wave 1 amplitude may be a robust proxy measure of age-related CS with/without accounting for OHC loss.

##### Human Studies

Otologically normal older adult humans have consistently been shown to exhibit smaller ABR amplitudes for waves I to V compared to their younger counterparts (Rowe, 1978; Maurizi et al., 1982; Allison et al., 1983; Costa et al., 1991; Konrad-Martin et al., 2012; Grose et al., 2019; Johannesen et al., 2019; Grant et al., 2020). Figure 3B shows the ABR wave I amplitude as a function of age in five different human studies (redrawn from Bramhall, 2021). An age-related decrease in the ABR amplitude measured at 110 dB peSPL at low click rates (i.e., 11 clicks/second) has been estimated at 38, 43, and 34% reduction for waves I, III, and V respectively for audiometrically normal-hearing individuals. This translates into 9.5, 10.8, and 8.5% amplitude reduction per decade for waves I, III, and V respectively (Konrad-Martin et al., 2012). The authors accounted for age-related increases in the audiometric thresholds, and thus the reduction in ABR wave I may not be attributed to OHC loss.

Bramhall et al. (2015) investigated the effect of age on ABR wave I amplitude by recruiting 57 adults (35 females) aged 19–90 with average pure tone audiometric thresholds at 0.5, 1, 2, and 4 kHz ranging between −1.25 to 38.75 dB HL. The ABR wave I amplitudes obtained using a 4 kHz tone burst presented at 80 dB nHL at a rate of 13.3/second were not influenced by the sex of the participants in the statistical model. After controlling for audiometric threshold loss, ABR wave I amplitude was found to decrease by about 17.8% per decade. Buran et al. (2022) provided a re-analysis of the Bramhall et al. (2017) data (*n* = 64; age range: 19–35; summarized in Table 3). After the potential confounds of sex and OHC function (as reflected by distortion product otoacoustic emission levels) were accounted for, ABR wave I amplitude measured at 110 dB peSPL was found to decrease by about 6.1% per decade.

Carcagno and Plack (2020) attempted to minimize the contribution of basal cochlear generators to ABR wave I (Eggermont and Don, 1978), which may be reduced by the effects of age, by band-pass filtering the click stimulus at 0.35–3 kHz and by presenting the click in a high-pass masking noise of 3.5–8 kHz (study summarized in Table 4). The authors reported an age-related reduction in wave I amplitude when high-pass masking noise was employed, at a rate of 12% reduction per decade (ages of subjects ranged from 18–70 years), with clicks presented at 80 dB p-peSPL. However, no age-related reduction was seen at 105 dB p-peSPL. This is the opposite pattern to that expected based on CS affecting low-SR fibers. In contrast, they observed an age-related wave I reduction of 17% per decade when no masking noise was used at 105 dB p-peSPL click level (but no reduction at 80 dB p-peSPL) even when controlling for high-frequency hearing loss in the statistical model. This latter result is consistent with CS in high-frequency cochlear regions (i.e., above the 3.5 kHz cut-off of the high pass masker). It is worth highlighting that this sort of masking paradigm has not been investigated in animal models of CS, so this approach has not been validated.

**Table 4:** Summary of the findings of key studies that investigated the combined effects of aging and noise exposure on wave I of ABR in humans. Data reported were either explicitly mentioned in the manuscript text or were derived from the relevant figures in the respective publications using the online tool of WebPlotDigitizer version 4.5 (Rohatgi, 2021).

**Study**	**Participants**	**ABR Recording Parameters**	**Outcomes**	**Sex-specific findings**
Valderrama et al. (2018)	74 participants (37 females) aged between 29–55 years. 84% of participants had normal hearing thresholds defined as ≤ 20 dB HL from 0.25–6 kHz	108.5 peSPL clicks using two reference electrode montage setups: ipsilateral mastoid (high forehead (Fz)-Tp9/Tp10) and ipsilateral ear canal (high forehead (Fz)-TIP)	After controlling for sex, amplitudes of wave I of ABR were smaller by 43.1% and 43.4% for participants with the 10% highest lifetime noise exposure compared to participants with the 10% lowest lifetime noise exposure using both the Fz-Tp9/Tp10 and the Fz-TIP electrode configuration respectively. Given all participants with various noise exposures, noise exposure was a significant predictor of ABR wave I amplitudes using Fz-Tp9/Tp10 montage (*p* = 0.0038) and Fz-TIP montage (*p* = 0.0215) The authors did not control for multiple comparisons, and the effect of noise exposure on the ABR wave I amplitude would not stay significant if the alpha level was adjusted for multiple comparisons of outcomes obtained using both electrode montages The effect of age was not considered in the analysis of ABR wave I data in relation to lifetime noise exposure, however, the authors argued that the reduction in the ABR wave I amplitude could be at least partially explained by the fact that middle-aged participants who were involved in the study tend to have age-related smaller ABR wave I amplitudes compared to younger participants	Males exhibited smaller ABR wave I amplitude compared to females
Prendergast et al. (2019)	156 participants aged 18–60 with hearing thresholds ≤ 20 dB HL up to 4 kHz and ≤ 30 dB HL at 8 kHz	100 dB peSPL clicks using the reference electrode montage of right (Fz-M1) and left (Fz-M2) mastoids	Neither age nor noise exposure had statistically significant effects on ABR wave I amplitude (*p >* 0.05). The Pearson's correlation coefficient between ABR wave I amplitude and age was−0.08	The authors did not report differences in the ABR wave I amplitude in relation to the sex of participants nor did they control for it in their analysis
Johannesen et al. (2019)	94 participants (64 females) aged 12–68 with hearing thresholds ≤ 20 dB HL at 0.5–4 kHz and ≤ 30 dB HL at 6–8 kHz	90–110 dB peSPL clicks using the reference electrode montage of the high forehead (Mastoid (M)-Fz)	Older participants had significantly lower wave I growth rates (for males *p* = 0.034; for females *p* = 0.00013). No effect of noise exposure on wave I growth was found (for males *p* = 0.2; for females *p* = 0.83). However, there was a trend of non-significantly smaller ABR wave I growth rates as a function of noise exposure for males only	The correlation between age and ABR wave I growth rates were stronger (i.e., more negative) in females compared to males
Carcagno and Plack (2020)	102 participants from three age groups: young (aged 18–39), middle-aged (aged 40–59), and older adults (aged > 60). All participants had hearing thresholds <20 dB HL at 0.125–2 kHz and <40 dB HL at 4 kHz	High level (105 dB p-peSPL) and low level (80 dB p-pe SPL) click in quiet and in high pass masking noise. The reference electrode montages used were ipsilateral earlobe (high forehead HF – ipsilateral earlobe IERL) and ipsilateral tiptrode (HF- ipsilateral tiptroad ITPR)	The ratio of wave I amplitude at high to low click levels was significantly decreased as a function of age (but no noise exposure) by a mean of about 12.6% per decade for the in-quiet ABR condition For the ABR in-noise condition, Wave I amplitude decreased as a function of age (but no noise exposure) by a mean of about 9.5% per decade using the low-level stimulus	Before controlling for sex, ABR wave I amplitudes in both the quiet and high-pass noise conditions were significantly larger for females compared to males at high-level stimuli

#### Auditory Brainstem Response Wave I: Combined Effects of Noise Exposure and Aging

##### Animal Studies

Fernandez et al. (2015) reported that the ABR wave 1 amplitude in 88-week old CBA/CaJ mice exposed to the noise of 8–16 kHz at 100 dB SPL for 2 h at 16 weeks of age was 35, 65, and 80% smaller compared to 88-week old unexposed counterparts, 24-week-old young exposed animals, and 24-week-old young unexposed mice respectively. These findings imply that noise exposure at a young age in CBA/CaJ mice may cause a further reduction in the amplitude of the ABR wave 1 as animals become older (compared to unexposed aged counterparts). The authors have shown that a slower rate of IHC-ANF synapse loss as a result of aging has occurred in cochlear regions with the most CS due to noise exposure (compared to control cochleae without noise exposure). This is consistent with our saturative noise exposure-aging CS model which proposes the vulnerability of low- and medium-SR ANFs only. Nonetheless, this 35% decrease in the ABR wave 1 amplitude in exposed older mice (compared to unexposed older counterparts) may stem from the fact that the ABR wave 1 amplitude may be influenced by other noise- and age-related factors that were not controlled for such OHC and IHC loss.

Möhrle et al. (2016) reported that pre-noise-exposed middle-aged (6–10 months) and older (19–22 months) rats exhibited a 40% smaller amplitude of wave 1 compared to pre-exposed young (2–3 months) rats. However, no further significant decrease in the amplitude of wave 1 of ABR in post-exposed middle-aged and older rats was noted compared to their pre-exposed middle-aged and older subject counterparts (animals were exposed to 8–16 kHz broadband noise at 100 dB SPL for 2 h). The key difference in methodology between Fernandez et al. (2015) and Möhrle et al. (2016) is that the animals in the Möhrle et al. (2016) study were not exposed to noise and then aged. Rather, they were aged and then noise exposed. In line with the patterns of synapse loss across the different age groups in this study (as discussed earlier in the histopathological section), the authors hypothesized that, as most vulnerable ANFs are lost as a result of aging, little further reduction in the amplitude of wave 1 of ABR is seen when noise exposure is added to middle-aged and older animals. This is consistent with our saturative model of CS which suggests that when only low- and medium-SR ANFs are vulnerable to noise exposure and aging, less CS loss may occur once the majority of vulnerable IHC-ANF synapses have been lost.

Although Fernandez et al. (2015) and Möhrle et al. (2016) employed different rodent species, with major methodological differences as highlighted above, their findings shed light on the potentially different patterns of noise-induced CS when noise exposure occurs at a young or old age. These differences should inform future human studies investigating the interaction of aging and noise exposure.

##### Human Studies

The contribution of both noise exposure and aging to the amplitude of ABR wave I in humans with normal/near-normal hearing was investigated by some studies, which have reported mixed results. Table 4 summarizes the methods and outcomes of these studies. Only Valderrama et al. (2018) reported that lifetime noise exposure may exacerbate an age-related decrease in the amplitude of wave I of the ABR. In contrast, other studies which considered the effects of noise exposure and aging found no correlation between lifetime noise exposure and ABR wave I amplitude (Prendergast et al., 2019; Carcagno and Plack, 2020). Similarly, Johannesen et al. (2019) reported no significant correlation between lifetime noise exposure and ABR wave I amplitude growth.

Several explanations have been proposed to justify the lack of consistency in the findings of the ABR wave I in relation to detecting CS across the different human studies. For instance, Bramhall et al. (2019) stated that the between-subject factors, which are difficult to control in human research, include the type (e.g., recreational vs. occupational/firearm noise) and duration of noise exposure as well as the tools used to retrospectively quantify them. Moreover, it could be difficult to rule out the presence of CS in the human control groups recruited based on self-reports of lifetime noise exposure. This is because noise exposure history is usually quantified using self-report questionnaires that primarily rely on subjects' ability to recall their history of noise exposure, which may not be optimally reliable and accurate (Bramhall et al., 2019). Another major concern with regards to the use of the ABR wave I amplitude is its potential lack of sensitivity to detect CS in humans due to the possibility that low-and medium-SR ANF responses may not contribute to ABR wave I amplitude (Versnel et al., 1990; Bourien et al., 2014). Rather, high-SR ANF activity may primarily dominate the ABR wave I amplitude (Bourien et al., 2014).

It has also been hypothesized that a noise-induced decrease in the amplitude of wave I of the ABR in normal-hearing humans could be so marginal that the current ABR wave I techniques may not be sensitive enough to detect it (Hickox et al., 2017). Prendergast et al. (2018) estimated that the coefficient of variation (CoV) of the ABR wave I amplitude was comparable to the wave V amplitude (i.e., CoV < 0.35). This may be in favor of detecting the effect of noise exposure on the ABR wave I amplitude. However, if this variance does not directly relate to noise exposure, then many hundreds of participants may be needed to detect small noise-induced changes, even at a group level.

Both Prendergast et al. (2018) and Guest et al. (2019b) estimated that the amplitude of wave I in young normal-hearing adults exhibits high test-retest reliability (intraclass correlation coefficient of 0.85). So by assuming that humans exhibit a similar proportion of synapse loss as the non-human primates of macaque monkeys (i.e., up to 27%), a reduction in the ABR wave I amplitude should be evident in humans in longitudinal studies. However, data from guinea pigs suggests that some cochlear synapses damaged following noise exposure were partially repaired (Song et al., 2016). A similar effect could happen in humans, and thus ABR wave I amplitude recovers to some extent. This recovery may also be variable across humans, which adds a further source of variability in the measurement of ABR wave I amplitude in CS studies. It should also be noted that humans could exhibit different genetic susceptibility to noise- and age-related CS. Hence, this could be another major source of variability that may influence ABR wave I amplitude reductions.

Finally, since both noise exposure and aging are thought to be associated with worse hearing thresholds in the extended high frequency (EHF) range (Matthews et al., 1997; Somma et al., 2008; Liberman et al., 2016; Bramhall et al., 2017), ABR wave I amplitude reduction may be confounded by the involvement of basal high-frequency cochlear generators such that smaller ABR wave I amplitude is recorded secondary to basal OHC loss (Eggermont and Don, 1978). As discussed earlier, it is important to establish the extent to which hearing threshold loss affects ABR wave I reduction, especially at high stimulus levels, in order to determine the efficacy of ABR wave I amplitude as a proxy measure of CS in the presence of noise-induced or age-related threshold elevations.

### Auditory Brainstem Response Wave I:V Amplitude Ratio

In addition to the amplitude of wave I of the ABR, other electrophysiological objective metrics have been used to assess CS in both animal and human research. For instance, the ratio of ABR wave I amplitude to wave V amplitude (wave I:V amplitude ratio) is thought to reflect the compensatory central gain that is hypothesized to take place as a result of the ANF deafferentation (Schaette and McAlpine, 2011). As a result, the amplitude of wave V could remain the same (as a result of central neural compensation) or even increase (in case of over-compensation), hence reflecting increased neural activity at the level of the mid-brain where wave V is generated. This may therefore translate into tinnitus and hyperacusis in humans (Gu et al., 2012; Hickox and Liberman, 2014). A potential limitation with the use of ABR wave I:V amplitude ratio as a proxy tool to detect and quantify CS is that the degree of central gain in response to reduced peripheral input (as indicated by wave V amplitude) may vary. This means that two individuals with identical ABR wave I amplitudes could have different wave I:V ratios depending on the degree of central gain.

It is important to note that the wave I:V amplitude ratio was found to exhibit high test-retest reliability in young normal-hearing adults (Prendergast et al., 2018). This suggests that this synaptopathy metric is probably still worth considering in future research. However, as described above in the discussion of wave I amplitude, it is not clear whether the wave I:V amplitude ratio is sensitive enough to detect and quantify CS cross-sectionally.

#### Auditory Brainstem Response Wave I:V Amplitude Ratio: Noise Exposure

The effect of noise exposure on the ABR wave I:V amplitude ratio is inconsistent across the literature. On the one hand, a few studies documented evidence for the central gain hypothesis such that no change to the amplitude of wave V was found while the amplitude of wave I was decreased in young human and rodent subjects with a history of noise exposure (Schaette and McAlpine, 2011; Hickox and Liberman, 2014; Bramhall et al., 2017). Megarbane and Fuente (2020) reported that a smaller wave I:V amplitude ratio is associated with worse SPiN performance (which is considered as a potential perceptual consequence of CS) in one ear only of audiometrically normal young adults with variable self-reported SPiN abilities. On the other hand, Guest et al. (2017) and Prendergast et al. (2017a) reported no evidence of a smaller wave I:V amplitude ratio in noise-exposed young normal-hearing human subjects compared to controls with minimal noise exposure. Grose et al. (2017) found a significantly smaller wave I:V amplitude ratio in subjects with high noise exposure compared to low-noise control subjects. However, the reduction in wave I:V amplitude ratio was not correlated with tinnitus, and primarily occurred due to a reduction in wave I amplitude alongside no statistically significant change in wave V amplitude.

#### Auditory Brainstem Response Wave I:V Amplitude Ratio: Aging

In older CBA/CaJ mice with already documented basal OHC loss, Sergeyenko et al. (2013) reported a decreased amplitude of wave 1 of ABR with no evidence for reduced wave 5 amplitude, thus the authors suggested that the ratio of wave 1:5 amplitudes may decrease as a function of age. Verhulst et al. (2016) predicted that high-frequency sloping sensorineural hearing loss, typically accompanying ARHL (and potentially associated with noise exposure), may contribute to a smaller ABR wave I:V amplitude ratio when ABR click stimuli are used. This is because damage to basal cochlear generators may reduce wave I amplitude but have a much smaller impact on the amplitude of wave V (Eggermont, 1976; Eggermont and Don, 1978; Verhulst et al., 2016).

Normal-hearing older human adults were found to exhibit a diminished wave I:V amplitude ratio compared to their younger counterparts (Grose et al., 2019). Likewise, Carcagno and Plack (2020) reported no age-related decrease in the amplitude of wave V evoked using 105- and 80- dB p-peSPL clicks in quiet. In contrast, when clicks were presented at 80 dB p-peSPL with high-pass masking noise, the median of wave V reduction was estimated at 14% per decade. Interestingly, the changes in the ABR wave I and V amplitudes reported by Konrad-Martin et al. (2012) as indicated in Figure 3B show constant age-related decline in the amplitudes of both waves I and V evoked using 110 dB p-peSPL clicks in quiet. The data by Konrad-Martin et al. (2012) are therefore inconsistent with those reported by Grose et al. (2019) and Carcagno and Plack (2020) in quiet, and go against the hypothesis that a central compensation secondary to age-related peripheral neural deafferentation results in little change or even enhanced ABR wave V amplitude secondary to aging.

#### Auditory Brainstem Response Wave I:V Amplitude Ratio: Combined Effects of Noise Exposure and Aging

Möhrle et al. (2016) reported that after young and middle-aged rats were exposed to moderately loud noise, wave 1 amplitude significantly decreased while wave 5 amplitude remained intact in both age groups. Following a similar noise exposure pattern in older rats, both wave 1 and wave 5 amplitudes were reduced, which may indicate a decreased neuronal gain as a result of central auditory aging. These findings may explain the reduced ABR wave V amplitudes reported by Konrad-Martin et al. (2012) who tested military veterans (who were likely exposed to significant firearm noise), in that the ABR wave I:V amplitude ratio could be affected by central aging, apart from CS itself.

Recent human studies measured the wave I:V amplitude ratio as a function of age while taking into account noise exposure history (Valderrama et al., 2018; Prendergast et al., 2019; Carcagno and Plack, 2020). These studies found no evidence for reduced wave I:V in middle-aged and older adults. It is worth pointing out that Valderrama et al. (2018) reported that middle-aged subjects with tinnitus had a statistically significantly lower wave I:V amplitude ratio compared to their non-tinnitus counterparts. However, the authors did not take into account the extent of audiometric threshold loss in their analyses, which could at least partially account for lower wave I:V amplitude ratios. These mixed findings add further uncertainty to whether the combined effects of aging and noise exposure result in CS-related compensatory central gain, and thus perceptually translate into tinnitus in humans.

### Summating Potential to Action Potential Ratio

#### Animal Studies

The SP:AP ratio has also been used as a metric of CS. The normalization of the auditory nerve AP (related to wave 1 of ABR) to the SP of hair cells is hypothesized to help in distinguishing presynaptic and postsynaptic damage at the IHC-ANF synapse (Sergeyenko et al., 2013). In aging CBA/CaJ mice with documented synapse loss, a large SP:AP ratio was found after age-related OHC loss was accounted for statistically. CS, in the absence of OHC loss, may hence compromise AP of the auditory nerve, while the SP remains intact (Sergeyenko et al., 2013).

#### Human Studies

In human studies, the rationale for the use of the SP:AP ratio is to control for possible sources of measurement variability, such as differences in head anatomy (Liberman et al., 2016). Liberman et al. (2016) found that the SP:AP ratio was increased in noise-exposed young normal-hearing adults compared to low-noise controls, although this was primarily due to greater SP rather than smaller AP. Similarly, Grant et al. (2020) reported increased SP and decreased AP in audiometrically-normal adults with the worst word recognition scores (as defined by the lower 25^th^ percentile of word recognition scores) compared to their best-performing counterparts (i.e., those with the highest 75^th^ percentile of word recognition scores). Chen et al. (2021) studied the SP:AP ratio in older adults with a confirmed age-related threshold elevation. The authors found that AP amplitudes were significantly reduced in participants with SP:AP ratios that were deemed abnormal (i.e., ≥ 34%) while the SP amplitudes were similar across the normal and abnormal SP:AP groups. These findings provide evidence that CS may occur as part of ARHL.

It is worth highlighting the poor test-retest reliability of the SP:AP metric reported by Prendergast et al. (2018), at least for the click level of 115.5 dB peSPL tested in that study. Hence, the SP:AP ratio may not be reliable enough to determine the combined effects of aging and noise exposure on CS. Additionally, the use of SP:AP metric in older adults might be complicated by age-related hair cell loss, which will require careful control, as performed by Sergeyenko et al. (2013) in their mouse study. Finally, it may be worth considering the approach proposed by Kamerer et al. (2020) in future studies. This method employs validated Gaussian functions to estimate the SP and the AP and is thought to provide a more reliable measure than visual inspection and determination (Kamerer et al., 2020).

### Envelope Following Response

The EFR is an objective auditory evoked potential characterized by neural responses that are phase-locked with the stimulus envelope modulation (Dolphin and Mountain, 1992). EFRs elicited with high-level stimuli with low modulation depths and high-frequency envelopes are thought to be sensitive to CS (Bharadwaj et al., 2014). This is because saturated high-SR fibers do not phase lock when presented with such stimuli, but low-SR fibers do (Bharadwaj et al., 2014, 2015; Shaheen et al., 2015; Verhulst et al., 2018b). Consequently, EFRs may be more sensitive to CS than ABR wave I amplitudes, not only because ABR measures are highly variable in humans and thus difficult to control for, but also because EFRs reflect phase locking to temporal envelopes in which low-SR fibers are strongly involved (Bharadwaj et al., 2014). Conversely, the computational model provided by Encina-Llamas et al. (2019) showed that the levels typically used to elicit EFRs (i.e., 70–80 dB SPL) may not be very specific to low-SR ANFs since, at these high intensities, the EFR responses are dominated by basal off-frequency high-SR ANFs that have not yet reached saturation. The computational model showed a minimal effect of subclinical OHC loss (which typically is associated with normal audiogram) on EFR amplitudes using the stimuli commonly presented at 70–80 dB SPL.

More recently, Vasilkov et al. (2021) provided evidence that the use of a stimulus with a rectangular envelope, with modulation rate, modulation depth, and duty cycles of 120 Hz, 95 and 25% respectively, presented at a fixed root mean square level of 70 dB SPL, may provide more sensitivity to CS while being minimally affected by co-existing OHC loss compared to sinusoidally amplitude-modulated tones that are commonly used. Moreover, Mepani et al. (2021) assessed the correlation between word recognition scores (words were presented in background noise) and EFR amplitudes using sinusoidally vs. rectangular-modulated carrier tones in otologically-normal adults aged 18–63. The sinusoidally amplitude-modulated tones were presented at 85 dB SPL using carrier frequencies of 1 or 8 kHz and were 100% amplitude-modulated at modulation frequencies of 128 or 750 Hz. The rectangular-modulated carrier tones were presented at 70 dB SPL at a modulation frequency of 120 Hz with a 25% duty cycle and 100% modulation depth. The word recognition scores were significantly positively correlated with EFR amplitudes evoked using rectangular-modulated tones, but not with sinusoidally modulated tones.

#### Envelope Following Response: Noise Exposure

##### Animal Studies

Shaheen et al. (2015) employed moderate stimulus levels (up to 90 dB SPL) with a carrier frequency of 11.3 kHz and 32 kHz and modulation frequencies ranging from 0.4–1.99 kHz to elicit EFRs in CBA/CaJ mice. EFR amplitudes were significantly reduced (by up to 55%) in noise-induced synaptopathic mice compared to non-synaptopathic controls at modulation frequencies near 1 kHz. For these high modulation frequencies, the EFR is thought to originate from the auditory nerve. This reduction, however, was not as large at lower modulation frequencies.

##### Human Studies

In humans, since EFRs obtained using a 1 kHz modulation frequency exhibit smaller amplitudes than in animal studies, lower modulation frequencies are often used which are thought to reflect neural generators from the midbrain rather than from more peripheral sources (Bharadwaj et al., 2015). For instance, Bharadwaj et al. (2015) assessed EFRs in young normal-hearing adults using a 4 kHz carrier tone modulated at 100 Hz, at a fixed level of 75 dB SPL with different modulation depths, presented in notched noise to restrict the cochlear region associated with the response. Subjects who showed the greatest decrease in EFR amplitude as a function of decreasing the modulation depth of the stimuli from 0 to −8 dB had the worst behavioral amplitude modulation thresholds (r = 0.53, *p* = 0.008). Moreover, the group of subjects who reported high past noise exposure had marginally significantly steeper positive EFR slopes (i.e., the slope of the line fit of EFR magnitudes in relation to modulation depths) compared to the low noise group (*p* = 0.034).

More recently, Bramhall et al. (2021) measured EFR amplitude in young audiometrically normal military veterans and non-veterans using a 4 kHz sinusoidally amplitude-modulated carrier tone presented at 80 dB SPL. The authors found that EFR amplitudes were 2.7, 2.5, and 3.4 dB smaller in the military veteran high-noise group at 100, 63, and 40% modulation depths respectively compared to the non-veteran control group. After adjustment for sex and OHC function, as reflected by the average distortion-product otoacoustic emission levels at 3–8 kHz, smaller EFR amplitudes were found at all modulation depths in high-noise military veteran male and female participants compared to their non-veteran counterparts.

Paul et al. (2017b) presented a 5 kHz carrier tone modulated at 86 Hz (with 0 dB modulation depth) at 75 dB SPL to two groups of young normal-hearing 18- and 19-year-old adults with and without significant noise exposure history. EFRs were measured both in quiet and in narrow band noise (NBN). The authors found reduced EFR magnitude for the high noise group compared with the low noise group. In a correction to the findings in the original publication, Paul et al. (2018) subsequently reported no statistically significant differences in the EFR amplitudes between the low and high noise groups across all measurement conditions (*p* > 0.05). Further studies such as those by Grose et al. (2017), Guest et al. (2017, 2018), Prendergast et al. (2017a) and Carcagno and Plack (2020) failed to find any significant relation between EFR amplitudes and lifetime noise exposure, tinnitus, or listening difficulties in young audiometrically normal adults. For the relation between EFR amplitudes and lifetime noise exposure, Grose et al. (2017) reported a *p*-value of 0.0664, while Guest et al. (2017) noted a correlation coefficient (r) of 0.01 between lifetime noise exposure and EFR amplitudes (*p* = 0.94). Prendergast et al. (2017a) found that the correlation coefficient (r) between lifetime noise exposure and EFR amplitudes obtained using 262 Hz pure tones was 0.08 (*p* > 0.05), while r was −0.16 (*p* > 0.05) when EFRs were elicited by 4 kHz pure tones. Guest et al. (2017) found that the tinnitus group had non-significantly lower EFR amplitudes than the control group (*p* = 0.1). Finally, Guest et al. (2018) reported similar EFR amplitudes across two groups of audiometrically-normal adults with and without listening difficulties (*p* = 0.99).

Paul et al. (2017a) assessed EFRs in young normal-hearing adults with and without chronic tinnitus using a 5 kHz carrier tone modulated at 85 Hz and presented at 75 dB SPL at three modulation depths of 0 dB (in quiet and in NBN), −2.5 dB with NBN, and −6 dB with NBN. In an erratum to the original publication, although no statistically significant difference in EFR amplitude was found between the tinnitus and control groups (*p* = 0.207), there was a trend toward lower EFR amplitudes for the tinnitus group compared to the control group (Roberts et al., 2018).

Other human studies based on computational simulation models of the peripheral and central auditory system predicted reduced EFR amplitudes in synaptopathic normal-hearing listeners (Verhulst et al., 2018a,b). The decreased EFR amplitudes were significantly associated with poor performance on psychoacoustic amplitude modulation tasks (*p* < 0.05; Verhulst et al., 2018a,b). Given the mixed findings using low modulation frequency stimuli in human studies, it is not clear whether the EFR at these frequencies is sensitive to noise-induced CS.

#### Envelope Following Response: Aging

##### Animal Studies

Progressive age-related CS has been associated with decreased EFRs to 1,024 Hz amplitude-modulated tones in older CBA/CaJ mice (Parthasarathy and Kujawa, 2018). This aging-EFR correlation was found significant across different tone levels and modulation depths. At lower modulation rates, which are dependent on more basal generators, decreased EFRs in older adults may arise not only from peripheral synapse loss but also from age-related deterioration in the central auditory system due to neural fiber loss and demyelination (Walton, 2010; Bharadwaj et al., 2014; Parthasarathy and Kujawa, 2018).

Lai et al. (2017) measured EFR amplitudes in young and aged Fischer-344 rats, using 8 kHz carrier tones modulated at frequencies of 45, 128, and 456 Hz and modulation depths ranging from 3.125% (−30 dB) to 100% (0 dB). The authors accounted for age-related peripheral hair cell and neural degeneration, which may manifest as poorer central neural responses, by adjusting the EFR stimulus level presented to the age groups so that the ABR amplitudes for these levels were similar. After this peripheral activation matching, the authors reported enhanced EFR amplitudes at 100% modulation depth (but not at 25% modulation depth) in the aged animals compared to their young counterparts. This was found when tones were modulated at 16–90 Hz (which are thought to generate EFRs originating from central auditory neural generators) were presented at 85 dB SPL. This age-related EFR amplitude enhancement suggests that older subjects had increased compensatory central gain as a result of decreased peripheral ANF neural activity.

To emphasize the differences in EFR while taking into account age-related central gain, the authors performed an additional “central” activation matching to the EFR stimuli. This was done by measuring the EFR amplitudes of old rats using 85 dB SPL tones that are 100% amplitude modulated at 45, 128, and 256 Hz with a carrier frequency of 8 kHz (which would stimulate the cochlear region with the least age-related changes in hearing thresholds). The median EFR amplitude in aged rats for each of the amplitude-modulated tones was measured. The authors then identified the EFR stimulus intensities to be used in the cental matching by measuring the EFR amplitudes in young rats using sinusoidally amplitude-modulated tones presented at 85–60 dB SPL (in 5-dB descending steps). The EFR stimulus intensity that produced equivalent central activation across the young and older rats was subsequently employed in EFR amplitude measurements. For both types of peripheral and central matching independently, no significant age-related differences in EFR amplitudes at different modulation depths and frequencies between the young and aged animals were reported, which suggests that peripheral and central auditory temporal coding was not different between the two age groups.

##### Human Studies

In humans, Prendergast et al. (2019) employed four low-frequency tones of 240–285 Hz to modulate a carrier frequency of 4 kHz at an intensity of 80 dB SPL in young and middle-aged audiometrically normal (up to 4 kHz) adults. The authors reported that participants' age did not predict EFR amplitudes (adjusted r^2^ = −0.004, *p* = 0.495). Patro et al. (2021) measured EFR amplitudes in audiometrically normal adults using a carrier frequency of either 2 or 4 kHz modulated at a rate of 91.42 Hz presented either in quiet (70 dB SPL at modulation depths of −8 or 0 dB) or in notched-noise (presented at an overall level of 60 dB SPL at modulation depths of −8, −4, and 0 dB). For the 2 kHz carrier frequency, the oldest adults had significantly reduced phase-locking value (PLV) of the EFR at 0 dB modulation depth in quiet compared to their youngest counterparts (*p* = 0.048). The oldest group produced the lowest PLV compared to the middle-aged and youngest adult group for the carrier frequency of 4 kHz at modulation depths of 0 dB in quiet (*p* = 0.031) and −8 dB in noise (*p* = 0.009).

More recently, Vasilkov et al. (2021) found that EFR amplitudes evoked by rectangular modulated stimuli presented at 70 dB SPL at a modulation rate of 120 Hz, a modulation depth of 95%, and a duty cycle of 25%, were significantly reduced in older adults with suspected age-related CS (*p* < 0.0001). Moreover, the authors found that their single-unit ANF simulation model suggested that ANFs fired more synchronously with this type of EFR stimulus compared to the commonly used sinusoidally amplitude-modulated stimuli (Vasilkov et al., 2021).

#### Envelope Following Response: Combined Effects of Noise Exposure and Aging

Carcagno and Plack (2020) measured EFR amplitudes in young, middle-aged, and older adults using two carrier tones of 0.6 and 2 kHz, modulated at around 100 Hz using two modulation depths of 100 and 70%, embedded in pink noise (to minimize the contribution of high-SR fibers) and using band-pass noise at 3–8 kHz (to minimize the contribution of high-frequency cochlear regions). The authors reported a significant age-related reduction in EFR amplitudes using a 0.6 kHz carrier at both modulation depths, while no effect was noted for the 2 kHz carrier at either modulation depth. No correlation between EFR amplitudes and lifetime noise exposure was found for either 0.6 or 2 kHz carrier tones. These findings are consistent with earlier studies such as those by Leigh-Paffenroth and Fowler (2006), Grose et al. (2009), and Garrett and Verhulst (2019) which documented an age-related decline in electrophysiological measures of phase-locking at subcortical levels using modulation rates of about 100 Hz.

Given the above studies, there is some evidence that aging may degrade EFR amplitudes, potentially due in part to the deterioration of central auditory pathways in older adults. However, the evidence on the effect of noise exposure on EFRs has been generally mixed and inconclusive. It is not yet clear whether EFRs are sufficiently sensitive, at least using the currently used research paradigms in humans, to capture CS and peripheral ANF loss. This is because human studies employed much lower modulation frequencies to elicit EFRs, unlike animal studies which mainly used higher modulation frequencies that are believed to reflect the function of more peripheral auditory neural generators (Parthasarathy and Kujawa, 2018). Moreover, EFR amplitudes in the aged population may be influenced by enhanced central gain, central neural dysfunction, and high-frequency cochlear damage, which may add further ambiguity to identifying CS in the low–mid-frequency range (Lai et al., 2017). Furthermore, Hesse et al. (2016) suggest that EFRs could be primarily mediated by high-SR rather than low-SR fibers at high levels and may not hence be effective in the search for low-SR fiber loss.

### Middle Ear Muscle Reflex

The MEMR, which in clinical terms is known as acoustic reflex (AR), is an objective measure of change in middle ear immittance that occurs as a result of an efferent feedback mechanism to the middle ear stapedial muscle in response to intense acoustic stimulation. Low- to medium-SR type I fibers may be involved in the afferent branch of the MEMR pathway (Kobler et al., 1992). Two types of MEMR approaches have been used in CS research: the standard tonal probe approach and the wideband probe approach. The standard tonal MEMR probe approach is widely used in clinical settings and measures middle ear admittance at one probe tone of 226 Hz or 1,000 Hz (Schairer et al., 2013). In contrast, the wideband probe MEMR determines middle ear admittance, power reflectance, and absorbance over a broad frequency range typically between 0.25 and 8 kHz (Schairer et al., 2013). Prendergast et al. (2018) and Guest et al. (2019b) reported that the MEMR thresholds obtained using the standard tonal probe approach exhibited high test-retest reliability in young audiometrically-normal human adults. This provides some promise to using the MEMR in the search for CS in humans.

#### Middle Ear Muscle Reflex: Noise Exposure

##### Animal Studies

**Figure 4:**
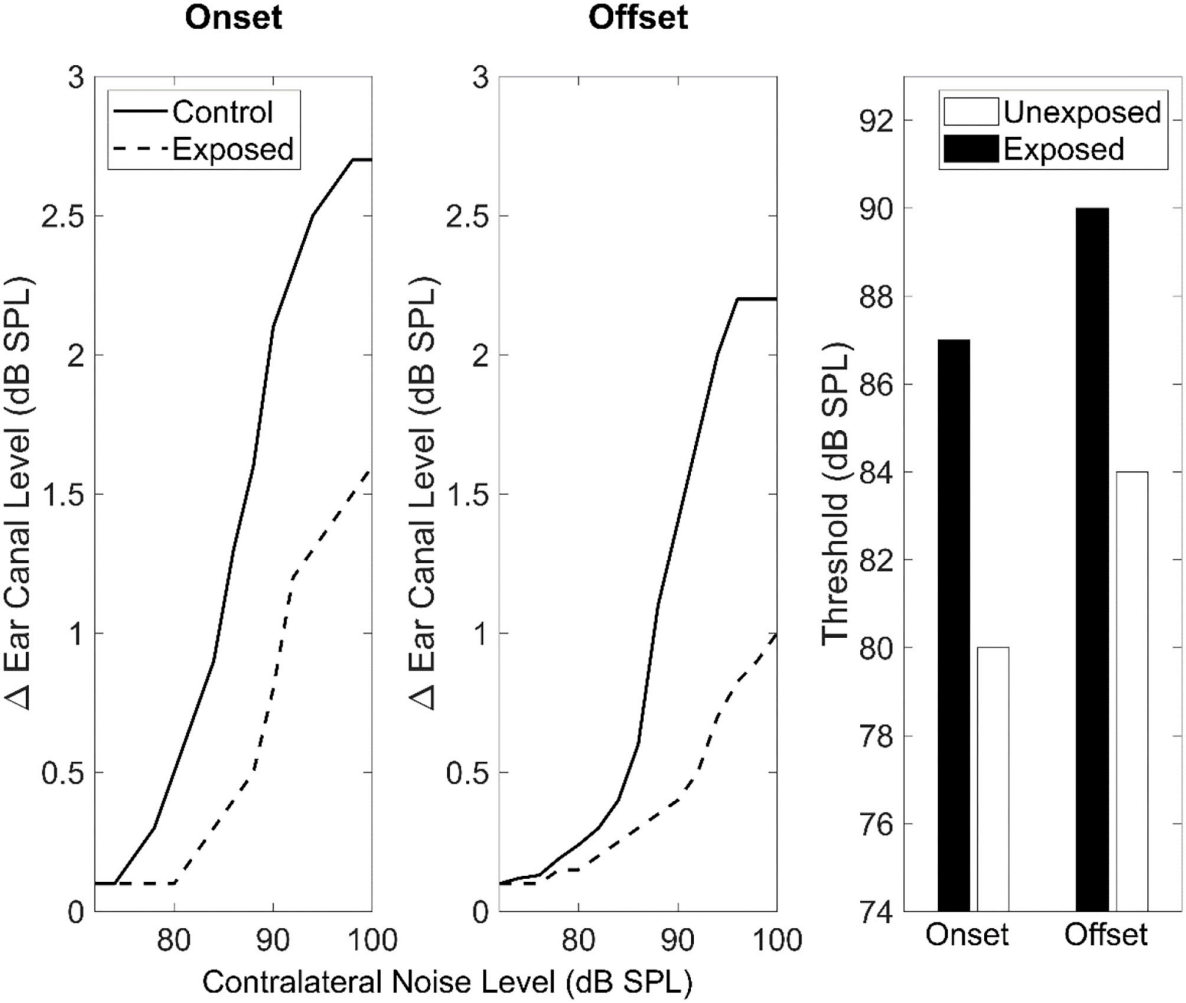
MEMR thresholds and growth functions (expressed as the difference in-ear canal SPL as a function of contralateral noise level) in noise-exposed and control mice measured at stimulus onset and offset. A wideband chirp covering a range of 4–64 kHz was presented contralaterally. This figure is redrawn from the data reported in panels A, B, and C of Figure 7 in Valero et al. (2016) using the online tool of WebPlotDigitizer version 4.5 (Rohatgi, 2021).

In mice with a histologically verified noise-induced CS, MEMR thresholds obtained using wideband probe and broadband elicitors were significantly increased while MEMR growth functions (i.e. MEMR magnitudes as a function of elicitor level) were considerably decreased at frequencies corresponding to the affected cochlear regions compared to non-synaptopathic areas (Valero et al., 2016, 2018). Therefore, the MEMR has been suggested as a good proxy for CS (Bharadwaj et al., 2019). Figure 4 shows a schematic representation of MEMR thresholds and growth functions in mice with verified CS compared to control mice respectively as measured at contralateral noise onset and offset (redrawn from Valero et al., 2016).

##### Human Studies

In humans, some recent studies have suggested a relation between MEMR amplitude and noise-induced CS. For instance, Shehorn et al. (2020) reported that high lifetime noise exposure is associated with lower ipsilateral broadband MEMR amplitude in normal-hearing young and middle-aged adults. Recently, Bramhall et al. (2022) measured the contralateral MEMR growth functions in 92 audiometrically-normal military veterans (who are typically exposed to firearm noise) and non-veterans aged 19–35 using a wideband probe and a broadband elicitor. The authors reported a trend of reduced MEMR growth functions in military veterans with high noise exposure compared to their non-veteran control counterparts. The mean difference in MEMR magnitude was lower by 0.29 dB in the veteran high noise group compared to the non-veteran control group. Other studies which involved normal-hearing young adults found a correlation between the presumed perceptual consequences of CS, such as poorer speech perception in noise and tinnitus, and reduced MEMR strength using the wideband probe approach (Wojtczak et al., 2017; Mepani et al., 2019; Shehorn et al., 2020). In contrast, Guest et al. (2019a) failed to find an association between MEMR thresholds (using the standard tonal probe and elicitors) and noise exposure, tinnitus, and coordinate response measure (CRM) SPiN thresholds. Moreover, Causon et al. (2020) failed to document a relationship between lifetime noise exposure in young normal-hearing subjects and MEMR thresholds and growth functions obtained using the clinical standard probe tone of 226 Hz and tonal elicitors. These negative findings may be potentially explained by the lack of sensitivity of the clinically MEMR protocol (which employs tonal elicitors and 226 Hz probe tone) to detect CS compared to the wideband probe and broadband noise elicitors employed by the other studies (Causon et al., 2020; Shehorn et al., 2020).

#### Middle Ear Muscle Reflex: Aging

Earlier studies suggest increased MEMR thresholds in normal-hearing older adults compared to their younger counterparts when measured by the standard clinical probe tone approach using broadband elicitors, but not low-to-mid frequency tonal elicitors (i.e., 0.5, 1, and 2 kHz), after controlling for the differences in audiometric thresholds (Silman, 1979; Gelfand and Piper, 1981). Wilson (1981) reported that older adults may show higher MEMR thresholds using the standard clinical probe tone approach, not only using broadband noise elicitors but also using tonal elicitors of 4 kHz and 6 kHz. Moreover, MEMR growth has been observed to decrease as a function of age (Thompson et al., 1980). In contrast, Unsal et al. (2016) found no differences in either the MEMR thresholds (obtained by the standard clinical probe tone approach) using 4 kHz tonal elicitors, or the MEMR decay, between older and younger adults. The correlation between MEMR thresholds/growth functions and aging in the above studies could be at least partially explained by age-related declines in central auditory neural pathways (Ouda et al., 2015), which need to be accounted for in the investigation of age-related CS using MEMR measures.

#### Middle Ear Muscle Reflex: Combined Effects of Noise Exposure and Aging

MEMR thresholds and growth functions using broadband noise elicitors may have promise as a measure of synaptopathy given the studies discussed above. However, it is not yet known whether lifetime noise exposure compounds the effect of age on MEMR strength.

## Behavioral Proxy Measures in Humans

In this section, the evidence from human studies on noise exposure, aging, and the combined effects of noise exposure and aging using behavioral proxy measures of CS will be discussed.

### Behavioral Proxy Measures in Humans: Noise Exposure

Based on the hypothesis that low- to medium-SR high threshold ANF fiber loss may affect speech perception at moderate-to-high levels (Liberman and Liberman, 2015), human studies have considered SPiN performance, and other proxy behavioral measures, concerning noise exposure in young normal-hearing adults. SPiN outcomes have been mixed and inconclusive (for reviews see Bramhall et al., 2019 and Le Prell, 2019).

Some studies have measured the effect of noise exposure on non-speech auditory psychoacoustic perceptual tasks in young normal-hearing adults. Measures such as interaural phase difference (IPD) discrimination, frequency and intensity difference limens, sound localization, and amplitude modulation detection have been used. Findings have been generally mixed and inconclusive. For instance, some studies reported that noise-exposed normal hearing adults exhibited poorer detection of temporal fine structure (e.g. discrimination of Gaussian noise from low-level noise with minimal envelope fluctuations) (Stone et al., 2008), worse amplitude modulation detection (Kumar et al., 2012; Stone and Moore, 2014; Verhulst et al., 2018b), and poorer IPD discrimination (Shehorn et al., 2020). In contrast, other studies failed to document a correlation between noise exposure and IPD discrimination, frequency, and intensity difference limens, sound localization, and amplitude modulation detection in young normal-hearing adults (Grose et al., 2017; Prendergast et al., 2017b, 2019; Yeend et al., 2017).

These mixed outcomes for behavioral proxy measures of CS in young noise-exposed humans with normal audiometric profiles could potentially be explained in three ways (Guest et al., 2018). Firstly, Noise-induced CS could not be as widespread in young normal-hearing adult humans as it is in rodent models. Secondly, the current behavioral measures in humans may not be particularly sensitive to CS. Based on signal detection theory, Oxenham (2016) showed that a synapse loss in humans up to 50% may not necessarily translate into measurable effects on behavioral tasks. Furthermore, the different behavioral tools used in human CS studies place variable sensory, perceptual, and central/cognitive demands (such as attention and memory), which likely contribute to inter-subject variability (Bramhall et al., 2019; DiNino et al., 2022). Thirdly, noise-induced CS in humans might not preferentially impair low- to medium-SR ANFs (as discussed in Section Histopathological and Neurophysiological Aspects: Noise Exposure). Moreover, low- to medium-SR ANFs might not have high thresholds in humans, consistent with evidence from non-human primates (Hickox et al., 2017). Hence, CS may not cause differential effects on performance as a function of stimulus level, as assumed by some measures.

### Behavioral Proxy Measures in Humans: Aging

Audiometrically normal/near-normal older adults with no cognitive decline have consistently been shown to exhibit poorer SPiN performance using different types of speech stimuli and competing background noises compared to their younger counterparts (Pichora-fuller et al., 1995; Kim et al., 2006; Füllgrabe et al., 2015; Vermeire et al., 2016; Babkoff and Fostick, 2017). Compromised temporal processing, which may arise due to age-related central neural degeneration as well as CS, has been suggested to explain the difference in performance (Gordon-Salant and Fitzgibbons, 1993; Füllgrabe et al., 2015; Babkoff and Fostick, 2017). It is worth highlighting that not all studies which found an age-related decline in SPiN performance controlled for cognitive performance when comparing outcomes to younger adults. While the effect of age-related CS on SPiN tasks cannot be ruled out, it is possible that age-related deterioration in the EHF (i.e., frequencies above the standard clinical range of 8 kHz) thresholds (Stelrnachowicz et al., 1989; Snell et al., 2002), central auditory processing (Caspary et al., 2008; Ouda et al., 2015) and cognitive decline (Humes and Dubno, 2009; Kamerer et al., 2019) may contribute to the observed differences. Moreover, the variability in audiometric hearing thresholds and OHC function was not controlled for in the studies investigating the age-related auditory perceptual deficits in audiometrically normal/near-normal adults as discussed above. This may partially influence SPiN/psychophysical outcomes in favor of the younger population, which generally has better OHC function and hearing thresholds.

Some studies have tried to isolate the effects of CS by measuring performance as a function of level, under the assumption that CS will differentially affect higher levels due to low- and medium-SR ANF loss. Prendergast et al. (2019) found that, for audiometrically normal adults, age did not predict performance on the CRM task in either the 40 and 80 dB SPL stimulus presentation conditions while hearing thresholds at 2 and 16 kHz were accounted for. However, older participants performed significantly better than their younger counterparts in the 40 dB SPL condition of the digits in noise (DIN) task while older age was associated with worse performance on the 80 dB SPL condition. This is in line with the hypothesis that older subjects with age-related CS affecting low- to medium-SR ANFs perform worse with higher-level SPiN stimuli, but not lower-level stimuli, compared to their younger counterparts. The effects of the hearing thresholds at 0.5 kHz and EHF threshold at 16 kHz were controlled for in two separate statistical models and they were shown to be significant predictors of DIN thresholds.

Carcagno and Plack (2021) measured CRM and DIN thresholds using low-pass filtered speech stimuli (at a cut-off frequency of 3 kHz) presented at low and high levels to audiometrically normal adults of various ages. The authors employed pink band-pass filtered noise at 3–8 kHz in both tasks to reduce the contribution of basal cochlear generators. No credible age-related declines were found in the CRM task (using both collocated and spatially separated maskers) or in the DIN task at either level. Likewise, Johannesen et al. (2019) attempted to isolate the effects of age-related CS by employing both sentences from the hearing in noise test (HINT) fixed at 65 dB SPL and disyllabic words at 50, 65, and 75 dB SPL, while the masking noise (which was either speech shaped noise SSN or the international female fluctuating masker IFFM) was varied adaptively. Authors found that age was a significant predictor of HINT thresholds using both SSN and IFFM maskers, but not of the disyllabic words in noise thresholds (using either masker). The effect of differntial speech level used in the HINT test was not a significant predictor of SPiN performance as a function of age, even after the variability in hearing thresholds across subjects is accounted for.

Patro et al. (2021) employed sentence target stimuli presented either as full-spectrum or lowpass filtered signal (presented at a fixed level of 75 dB SPL in both conditions) embedded in a speech masker of either the same or different F_0_. The proportion of correct scores was measured in two spatial conditions: co-located (i.e., target and masker at 0° azimuth) and non-colocated (target and masker at ±15° azimuth). A significant age effect was reported for both conditions of the full-spectrum and lowpass-filtered speech target embedded with the same/different F_0_ speech maskers, however, no significant interaction between the spatial condition and age group was found.

Age-related declines in performance in psychoacoustic tasks in audiometrically normal older adults are inconsistent across the literature. For instance, on the one hand, decreased performance on amplitude modulation tasks (He et al., 2008; Füllgrabe et al., 2015; Wallaert et al., 2016; Carcagno and Plack, 2021), IPD discrimination (King et al., 2014; Füllgrabe et al., 2015; Carcagno and Plack, 2021), gap detection thresholds for a tone in noise (Patro et al., 2021), and frequency discrimination (He et al., 1998; Clinard et al., 2010) has been found in older adults compared to their younger counterparts. On the other hand, data from Grose et al. (2019), Paraouty et al. (2016), Patro et al. (2021), Prendergast et al. (2019) and Schoof and Rosen (2014) (amplitude modulation detection), Carcagno and Plack (2021) and Patro et al. (2021) (low-frequency carrier IPD discrimination task), Prendergast et al. (2019) and Patro et al. (2021) (high-frequency carrier IPD discrimination task) and Bianchi et al. (2019) and Carcagno and Plack (2021) (for frequency discrimination) provide no evidence for age-related declines in these psychophysical tasks. This inconsistency in findings may be partly explained by the fact that not all studies accounted for the variability in hearing thresholds, EHF thresholds, cognitive factors, past musical training, as well as central auditory processing ability in the analysis of their psychoacoustic data.

A few studies have attempted to isolate the effects of age-related CS on psychoacoustic tasks by presenting the psychophysical stimuli at different levels such as those by Prendergast et al. (2019) and Carcagno and Plack (2021). Yet, the outcomes of these studies provide little evidence of poorer performance at higher stimulus levels.

### Behavioral Proxy Measures in Humans: Combined Effects of Noise Exposure and Aging

A few recent studies have attempted to evaluate the combined effects of aging and lifetime noise exposure on SPiN tasks. For instance, Valderrama et al. (2018) found that SPiN performance (using the high cue LiSN-S test) in young and middle-aged normal hearing adults was neither predicted by their age nor by their lifetime noise exposure. Similarly, Johannesen et al. (2019) showed that while noise exposure did not seem to influence the SPiN scores, older normal hearing subjects performed worse on a SPiN task involving words presented in steady and fluctuating noises compared to their younger counterparts. However, age (which ranged from 12 to 68 years in Johannesen et al., 2019 study) did not seem to influence the performance of participants in a different SPiN task involving sentences embedded in the same types of noises. Furthermore, Prendergast et al. (2019) and Carcagno and Plack (2021) reported that neither age nor lifetime noise exposure predicted the SPiN performance of subjects using the CRM task. However, the authors had conflicting findings concerning the effect of age using the DIN task, such that Prendergast et al. (2019) reported that older age was unexpectedly associated with better DIN thresholds at low stimulus levels while higher lifetime noise exposure was associated with better scores at high stimulus levels. In contrast, Carcagno and Plack (2021) found that neither age nor noise exposure had effects on DIN thresholds using their band-limited stimuli.

The evidence on the combined effects of aging and lifetime noise exposure on psychoacoustic tasks is sparse and inconclusive. Prendergast et al. (2019) and Carcagno and Plack (2021) have recently found that neither aging nor lifetime noise exposure was correlated with performance on a high-frequency carrier IPD task (Prendergast et al., 2019) and low-frequency carrier IPD task (Carcagno and Plack, 2021). Moreover, Carcagno and Plack (2021) found no interaction between lifetime noise exposure and aging on the amplitude modulation detection and frequency discrimination tasks. These inconsistent and mainly negative findings add further doubt to the sensitivity of these psychoacoustic tasks in detecting CS.

## Summary and Recommendations for Future Research

In summary, animal histopathological studies have shown that both noise exposure and aging result in a substantial, yet highly variable, degree of synapse and ANF loss across several species. Rodent studies on the combined effects of noise exposure and aging suggest that animals who experience intense noise exposure at a young age may exhibit substantial noise-induced CS, and then go on to exhibit further CS as they age. However, the impact of noise exposure on older animals tends to be reduced, suggesting a saturation-like effect.

In young adult humans, histopathological studies are still lacking on the effects of noise exposure on synapse loss. Recently, Wu et al. (2021) have confirmed noise-related ANF loss in middle-aged and older human subjects. With regards to aging, human temporal bone studies suggest an age-related loss of synapses and ANFs, but these could not ascertain whether the lost fibers were primarily low-to-medium-SR ANFs, as is the case in rodent models, due to the lack of methods for classifying ANFs based on their SR in humans. The current human temporal bone data seem to be consistent with a model that assumes that only a portion of synapses (perhaps those with low- and medium-SR ANFs) are vulnerable to aging and noise exposure. While noise exposure was associated with a reduction in ANFs for middle-aged adults, older adults, who had a reduced baseline number of ANFs, did not show an additional effect of noise exposure (Wu et al., 2021). There are two possible explanations for the observed effect: first, these older adults may have reached the maximum extent of synapse loss, due to the effects of age alone, thus no further CS has taken place due to noise exposure; alternatively, the older “unexposed” adults may have had considerable undocumented noise exposure that eventually resulted in a similar extent of CS compared to their “exposed” counterparts.

Animal studies have consistently shown that noise-induced and age-related synapse and ANF loss are related to reductions in objective metrics (i.e., ABR wave 1, EFR, and MEMR amplitudes). In humans, objective and behavioral measures have produced inconsistent outcomes in relation to noise-induced CS, with some studies showing effects consistent with CS and others not. It is worth pointing out that estimates of the effect of noise exposure on physiological proxy measures of CS vary, with some studies showing large effects and others showing small non-significant effects. Some of this variability may be due to variability in study design and the type of noise exposure (e.g., military noise vs. recreational noise) as discussed earlier. In contrast, age-related changes in objective (e.g., wave I of ABR, EFR, and MEMR) and behavioral metrics are generally consistent across the human literature. However, it is not clear whether these changes relate directly to the synapse loss or are brought about by the age-related changes that occur across the entire auditory neural pathways. Only a few behavioral studies have attempted to isolate the effects of CS by comparing outcomes across levels, and these have not shown any clear differential effects. Future research will also need to account for the age-related loss of basal hair cells when investigating electrophysiologic neural responses (e.g., wave I of ABR and EFR) as well as the effects of cognitive decline when measuring behavioral performance in older adults.

Most of the current evidence in humans is based on observational cross-sectional studies that involve proxy objective or behavioral measures. Future research may need to employ longitudinal study designs and focus on the development and employment of more sensitive objective and behavioral tools based on a gold-standard measure of CS in living humans that relies on more robust CS models derived from animal and human temporal bone data. In particular, wideband MEMR thresholds and growth functions when measured using broadband elicitors are promising as sensitive measures of CS in humans. It may also be critical to establish more sensitive estimation tools of lifetime noise exposure such as by developing noise exposure metrics validated to objective measures (e.g., dosimetry). The need to control for differences in genetic susceptibility to noise- and age-related CS may still be a challenge in future research studies.

Although we recognize that it may be difficult to disentangle and control for all the different factors that may influence peripheral neural auditory aging, we recommend that future research focuses on the effects of noise exposure and aging in combination, rather than in separation, by determining when in the human lifespan noise exposure has occurred and the rate of progression of CS in ARHL using both histopathological and proxy approaches. This could be potentially achieved by controlling for past exposure to ototoxic substances and carefully screening and accounting for pathologic history, particularly some common chronic conditions among older adults that may affect peripheral hearing such as diabetes, blood hypertension, as well as genetic factors that may accelerate ARHL. Longitudinal study designs may be particularly useful in this regard, for instance studying cohorts of humans who are noise-exposed in occupational settings, compared to controls with a quiet lifestyle.

## Author Contributions

All authors listed have made a substantial, direct, and intellectual contribution to the work and approved it for publication.

## Funding

This work was supported by an internal Ph.D. grant from the Faculty of Biology, Medicine, and Health at the University of Manchester, the Medical Research Council (MR/V01272X/1), and the NIHR Manchester Biomedical Research Centre.

## Conflict of Interest

The authors declare that the research was conducted in the absence of any commercial or financial relationships that could be construed as a potential conflict of interest.

## Publisher's Note

All claims expressed in this article are solely those of the authors and do not necessarily represent those of their affiliated organizations, or those of the publisher, the editors and the reviewers. Any product that may be evaluated in this article, or claim that may be made by its manufacturer, is not guaranteed or endorsed by the publisher.
